# Taxonomic diversity of fecal microbiota associated with different metabolic phenotypes in residents of Arkhangelsk, Northwestern Russia

**DOI:** 10.7555/JBR.39.20250258

**Published:** 2026-09-28

**Authors:** Anna Postoeva, Ekaterina Krieger, Anna Leontyeva, Alexander V. Kudryavtsev, Julia Galeeva, Dmitry Fedorov, Polina Kuzmichenko, Elena Ilina, Vadim Govorun

**Affiliations:** 1 Department of Hospital Therapy and Endocrinology, Northern State Medical University, Arkhangelsk 163000, Russia; 2 International Research Competence Centre, Central Scientific Research Laboratory, Northern State Medical University, Arkhangelsk 163000, Russia; 3 Department of Infectious Diseases, Northern State Medical University, Arkhangelsk 163000, Russia; 4 Research Institute for Systems Biology and Medicine, Department of Mathematical Biology and Bioinformatics, Moscow 117246, Russia

**Keywords:** gut microbiota, metabolic phenotypes, metabolic syndrome, obesity, Russia

## Abstract

The study assessed the taxonomic diversity and composition of gut microbiota in Arkhangelsk residents, Northwestern Russia, with varying metabolic statuses. A population-based cross-sectional "Know Your Heart" study (2015–2017, participants aged 35–69 years) included a health examination and gut microbiota analysis (*n* = 685). Participants were divided into four metabolic phenotypes: metabolically healthy non-obese (MHN), metabolically unhealthy non-obese (MUN), metabolically healthy obese (MHO), and metabolically unhealthy obese (MUO). Analyses were performed using RStudio software (version 4.2.0) with the vegan and phyloseq packages (version 1.42.0) for microbiota analysis. The sample was distributed across phenotypes as follows: MHN (47.7%), MUN (22.6%), MHO (10.4%), and MUO (19.3%). Beta-diversity analysis revealed significant differences in overall microbiome composition between MUO and MHN participants, while alpha-diversity did not differ significantly across phenotypes. The MHN group was characterized by a higher abundance of beneficial commensals such as *Christensenellaceae R-7 group*, *Ruminococcaceae UCG-005*, and *Eubacterium xylanophilum group*, which are taxa previously associated with metabolic health and longevity. In contrast, the MUO group showed an increased abundance of *Streptococcus salivarius* and *Negativibacillus*, taxa whose potential roles in metabolic health warrant further investigation. *Blautia* spp. emerged as a major hub in the microbiota of obese participants, consistent with its reported association with visceral fat. In conclusion, gut microbial composition differed across metabolic phenotypes, with the most notable taxonomic differences observed between MHN and MUO participants. Several taxa associated with metabolic health were enriched in the MHN group, whereas other taxa were more abundant in the MUO group. These findings suggest that distinct gut microbial signatures are associated with obesity and metabolic dysfunction, although the cross-sectional design precludes causal inference.

## Introduction

In 1997, the World Health Organization (WHO) officially declared obesity a global pandemic, recognizing its growing prevalence and health consequences^[1]^. Obesity is a major risk factor for several chronic diseases, including type 2 diabetes mellitus, cardiovascular disease, certain cancers, and non-alcoholic fatty liver disease, placing a significant burden on healthcare systems worldwide^[2]^.

The controversial theory known as the "obesity paradox" was described in the early 2000s, following the discovery of a protective role of obesity in patients with acute cardiovascular outcomes. However, this protective role has been debated and is likely explained by methodological issues^[3]^. Later, the concept of "metabolically healthy obesity" emerged, based on data showing no increased risk of cardiovascular outcomes among individuals with a high body mass index (BMI) and the absence of metabolic syndrome (MetS)^[4]^. Based on the presence or absence of obesity and MetS, four metabolic phenotypes were introduced to differentiate the health effects of the two conditions^[5]^.

One theory explaining metabolic status and obesity rests on the influence of gut microbiota, a complex ecosystem consisting of numerous bacterial populations, with approximately 10^11^ microorganisms per gram of feces^[6]^. Intestinal microbiota may play a role in the pathogenesis of obesity, as alterations in its taxonomic composition may be associated with energy balance, fat storage, and inflammation^[6]^. Many researchers agree that the microbiota in obese individuals exhibits high "energy harvesting efficiency", which means certain intestinal microbes are more efficient at breaking down complex polysaccharides into absorbable sugars^[7]^. Alterations in microbiota composition in obesity are characterized by a decrease in the diversity and abundance of dominant bacterial types, particularly *Firmicutes* and *Bacteroidetes*, and a reduction in their beneficial metabolites. Levels of certain intestinal metabolites, including fatty acids such as dodecanedioic acid, arachidic acid, and mevalonic acid, have been found to correlate with changes in microbiota composition and may play a role in obesity development^[8]^.

Several studies of gut microbiota in obese patients without insulin resistance have demonstrated a predominance of genera such as *Alistipes*, *Bifidobacterium*, *Eubacterium*, *Faecalibacterium*, *Ruminococcus*, and *Subdoligranulum*^[8–9]^. Similar studies have reported an increased abundance of *Ruminococcus*, various species of *Bacteroidetes*, and *Enterobacter* in patients with obesity and MetS, compared with individuals without metabolic disorders^[9–10]^.

We found a lack of population studies simultaneously investigating gut microbiota in four metabolic phenotypes defined by the presence or absence of obesity and MetS. Accordingly, the current study aimed to assess the taxonomic diversity and composition of gut microbiota in an adult population-based sample divided into these four phenotypes.

## Materials and methods

### Ethical considerations

The "Know Your Heart" (KYH) study was approved by the Institutional Review Board of the Northern State Medical University (NSMU) in Arkhangelsk (No. 01/01-15, 2015) and the Institutional Review Board of the London School of Hygiene and Tropical Medicine in London (No. 8808, 2015). The protocol for the current study was also approved by the ethics committee of NSMU (No. 9-11/21, 2021).

### Samples and data collection

In 2015–2017, the KYH cross-sectional study was conducted with a random population sample of adults aged 35–69 years from Arkhangelsk, Northwestern Russia (*n* = 2380). Participants underwent a standardized interview, physical examination, and provided blood and fecal samples for laboratory testing. Further details of the KYH study design have been published by Cook *et al*^[11]^.

Sociodemographic characteristics of KYH participants used in this study included age, sex, education (higher, incomplete secondary, and secondary), and occupation category according to the International Standard Classification of Occupations (ISCO) (high-skilled white-collar, low-skilled white-collar, high-skilled blue-collar, and low-skilled blue-collar). Several lifestyle characteristics were also considered. Smoking status was defined as non-smoker, former smoker, or current smoker. Drinking level (non-drinking, non-problem drinking, hazardous drinking, and harmful drinking) was assessed using a combination of the Alcohol Use Disorders Identification Test (AUDIT), the Cut down, Annoyed, Guilty, Eye-opener (CAGE) test, and self-reports of harmful drinking patterns as previously described^[12]^. Diet quality was defined using the Dietary Quality Score. Physical activity was measured using the European Prospective Investigation into Cancer and Nutrition (EPIC) questionnaire and categorized into four levels (inactive, moderately inactive, moderately active, and active). Previously diagnosed diseases (diabetes mellitus, cancer, kidney disease, arterial hypertension) and medications taken were self-reported as part of the interview. Physical examination included BMI assessment based on weight (kg) and height squared (m^2^). Blood pressure was measured three times, and the mean of the second and third measurements was analyzed.

Blood samples in the KYH were collected at least four hours after the last meal and centrifuged. The serum was then frozen at −80 ℃, shipped to the study laboratory in Moscow on dry ice (−50 ℃), and analyzed in a single batch. Levels of high-density lipoprotein cholesterol (HDL-C, mmol/L) and triglycerides (TG, mmol/L) were measured by enzymatic colorimetric assays, whereas C-reactive protein (CRP, mg/L) and glycated hemoglobin (HbA1c, %) were assessed by immunoturbidimetric methods (AU 680 Chemistry System; Beckman Coulter, Tokyo, Japan).

Participants provided fecal samples voluntarily, either at the clinic or by bringing them in later. If samples were collected at home, participants were instructed to store them at 4 ℃ and bring them to the clinic within 18 h of collection. Samples were obtained from 1013 participants, aliquoted into cryovials, and frozen at −80 ℃. Frozen samples were shipped to the laboratory in Moscow on dry ice, where DNA was isolated and stored at −20 ℃ until further analysis. A random subset of 728 samples was subjected to gut microbiota analysis.

Based on physical examination and laboratory tests, participants were divided into four phenotypes depending on the presence of obesity (defined as BMI > 30 kg/m^2^) and metabolic disorders. Metabolic disorders were defined as meeting two or more of the four MetS criteria (American Heart Association/National Heart, Lung and Blood Institute, 2009): (1) arterial hypertension (blood pressure ≥ 130/85 mmHg and/or use of antihypertensive medications); (2) low HDL-C (< 1.03 mmol/L in men and < 1.3 mmol/L in women); (3) elevated TG (≥ 2.1 mmol/L, as blood samples were non-fasting); and (4) diabetes mellitus (HbA1c ≥ 5.7% and/or use of antidiabetics and/or self-reported diagnosis with prescribed treatment)^[5]^. HbA1c was used instead of fasting glucose because participants were allowed to attend the examination either non-fasting or after fasting for four hours. Participants without MetS were considered "metabolically healthy". Accordingly, the four phenotypes were defined as follows: (1) metabolically healthy non-obese (MHN), (2) metabolically unhealthy non-obese (MUN), (3) metabolically healthy obese (MHO), and (4) metabolically unhealthy obese (MUO).

Analysis for the current study included 685 KYH participants with gut microbiota data and all variables required for phenotype assessment. The metadata used in this analysis are provided in ***Supplementary Table 1***.

### 16 S rRNA sequencing

Total DNA was isolated from 1 mg of each collected fecal sample using the MagMAX DNA Multi-Sample Ultra 2.0 Kit (Roche Diagnostics, Mannheim, Germany) and a KingFisher Flex automated isolation station (Thermo Fisher Scientific, Waltham, MA, USA). Genomic libraries were prepared and sequenced according to the 16S metagenomic sequencing library preparation protocol for MiSeq (Illumina, San Diego, CA, USA). Each sample was processed using the Tersus PCR kit (Evrogen, Moscow, Russia). Primers 341F and 801R were used to amplify the V3-V4 region of the 16S rRNA gene. Unique combinations of indexing primers, analogs of the primers from the Nextera XT Index kit v2, were used to barcode samples. The sequencing procedure was performed on a MiSeq Sequencing System (Illumina) using the MiSeq Reagent Kit v2 (Illumina) according to the manufacturer's instructions. Samples were sequenced in batches, and batch was included as a covariate in the analysis. There were four batches in the study. The distribution of samples across batches is shown in ***Supplementary Fig. 1***. Quality control metrics, including read depth and rarefaction curves, are illustrated in ***Supplementary Fig. 2***.

### 16 S rRNA data processing

Residual adapters were removed using Trimmomatic (version 0.36)^[13]^, and quality filtering of reads was performed with the filterAndTrim function from the DADA2 package^[14]^. Denoising, merging, and chimera removal were carried out with DADA2 software (version 1.24.0) using the following parameters: for learnErrors, nbases = 1e+09, randomize = TRUE, MAX_CONSIST = 2; for dada, pool = TRUE; for mergePairs, minOverlap = 18; for removeBimeraDenovo: allowOneOff = FALSE, method = "consensus". Taxonomy annotation was performed against the SILVA reference database (version 138)^[15]^. The Phylogenetic Investigation of Communities by Reconstruction of Unobserved States (PICRUSt2) tool (version 2.5.1) was used to predict functional abundances from 16S data and a reference genome database with stratified output. Kyoto Encyclopedia of Genes and Genomes (KEGG) orthologs data after PICRUSt2 were agglomerated at the KEGG pathway level using the HMP Unified Metabolic Analysis Network (HUMAnN version 3.8)^[16]^.

### Statistical analysis

Statistical analyses were performed using RStudio software (version 4.2.0; Posit, PBC, Boston, Massachusetts, USA). The vegan^[17]^ (version 2.6-6.1) and phyloseq packages^[18]^ (version 1.42.0) were used for microbiota analysis. To remove low-abundance amplicon sequence variants (ASVs), the core_members function from the microbiome^[19]^ package was applied with a detection threshold of one and a prevalence threshold of 5%. This filtering process resulted in 567 ASVs and 141 genera for downstream analysis.

All pairwise comparisons of biochemical parameters between phenotypes and microbiota samples were performed using the compare_means function from ggpubr^[20]^ (version = 0.6.0, method = wilcox.test, p.adjust.method = "BH").

To evaluate whether the distribution of the metadata variables differed significantly among the studied phenotypes, a chi-square test was employed.

Associations between microbial taxa and host, as well as pathway-level functional profiles and host variables, were identified using permutational multivariate analysis of variance (PERMANOVA). The adonis2 function from the vegan package was employed, with 1000 permutation tests performed on the Bray–Curtis distance matrix.

The diversity of the bacterial microbiota was assessed using alpha-diversity (α-diversity) indices, including the Shannon, Simpson, and Chao1 indices, at the genus and ASV levels. Beta diversity (β-diversity) was assessed by principal coordinates analysis (PCoA) based on Bray–Curtis dissimilarities. Differences in overall community composition among phenotypes were assessed using analysis of similarities (ANOSIM), whereas PERMANOVA implemented with the adonis2 function was used to evaluate associations between microbiota composition and host or technical variables.

The Dirichlet multinomial mixtures (DMM) approach from the package DirichletMultinomial (version 1.40.0)^[21]^ was applied to a genus-level matrix of samples from all participants to stratify the joint microbiota dataset into community types. The association between phenotypes, batch, and microbial clusters (enterotypes) was assessed using the chi-square test.

The co-abundance networks of microbial ASVs were generated using the SParse InversE Covariance Estimation for Ecological Association Inference (SPIEC-EASI version 1.1.3)^[22]^ algorithm with the Meinshausen-Bühlmann method for detecting correlations from the NetCoMi package (version 1.1.0)^[23]^. The following parameters were utilized: 100 subsamples, 25 lambda iterations, and a minimum lambda value of 0.001. From the resulting network, clusters of co-abundant ASVs were identified using the Louvain method. Eigenvector centrality and degree centrality were used to define hubs/keystone taxa (nodes with a centrality value above the empirical 95% quantile). NetCoMi used a bootstrap approach for calculating the *P*-value. Only statistically significant correlations were retained for analysis.

To assess differential abundance at the genus, ASV, and pathway levels with a detection threshold of 10 and a prevalence threshold of 5%, we employed an ensemble of statistical models using the Differential Expression analysis for Sequencing data (version 2; DESeq2)^[24]^ and Multivariate Association with Linear Models package (version 2; MaAsLin2)^[25]^ in R. Both tools were used with the following formula: ~ phenotype + age + sex + batch, where the variable of interest was "metabolic phenotype" and the other variables were used as covariates. These covariates were selected based on their contribution to explaining diversity (as assessed by PERMANOVA), their significance in chi-square tests, and their overall biological relevance. The DESeq2 analysis was conducted using the Wald test with a parametric fitting approach and the 'poscounts' method for size factor estimation, which avoids the limitations of pseudocount addition and provides more accurate normalization of sparse count data by excluding zero-count features and using robust median-based calculations. In DESeq2, the default Wald test with Benjamini–Hochberg *P*-value correction was used to assess the statistical significance of log_2_(fold change). For MaAsLin2, we employed compound Poisson linear models (CPLM) and linear models (LM) with multiple parameter combinations in the format model + normalization + transformation: CPLM + total sum scaling (TSS) + NONE, CPLM + cumulative sum scaling (CSS) + NONE, CPLM + trimmed mean of M-values (TMM) + NONE, LM + centered log ratio (CLR) + NONE, and LM + TSS + logarithmic transformation (LOG). After all features were evaluated, *P*-values were adjusted for multiple hypothesis testing with the Benjamini–Hochberg correction, and the "coef" in MaAsLin2 represented the regression coefficient in the model. Finally, only taxa and pathways that were significantly differentially abundant in at least three out of six models were selected for downstream analysis. Only taxa with *P*_adjusted_ < 0.05 were selected. To assess biological significance beyond statistical significance, the mean absolute effect size (|mean effect size|) was calculated across all models for each differentially abundant taxon, providing a measure of the overall magnitude of abundance differences between groups.

Pearson correlation analyses were conducted between biochemical parameters and CLR-transformed abundances of differentially represented taxa using the cor.test function from the stats package (R version 4.2.0)^[26]^. *P*-values were adjusted for multiple testing using the Benjamini–Hochberg procedure.

### Data and code availability

Sequencing reads obtained for samples were deposited in the National Center for Biotechnology Information (NCBI) as BioProject PRJNA1225157. R code used for the analysis is available at the following link: https://github.com/META-SBM/16s_Arkhangelsk_paper. Researchers may apply for access to other KYH data. See data access regulations and instructions at https://metadata.knowyourheart.science. All data requests will be guided by the protection of personal information, the confidentiality agreement with the participants, and participants' informed consent.

## Results

### Study population

The current study included 685 participants aged 35–69 years (401 women and 284 men). The main characteristics of the phenotypes are shown in ***Fig. 1***. The MHN phenotype was observed in 327 participants (47.7%), MUN in 155 participants (22.6%), MHO in 71 participants (10.4%), and MUO in 132 participants (19.3%) (***Fig. 1***). The proportions of females were 57.8%, 51.0%, 69.0%, and 63.6%, respectively (*P* = 0.043). Phenotypes showed no significant differences in smoking status (*P* = 0.203) and drinking level (*P* = 0.903). Biochemical parameters including TG, HDL-C, HbA1c, and CRP differed between the four groups as expected (***Fig. 1B***).

**Figure 1:**
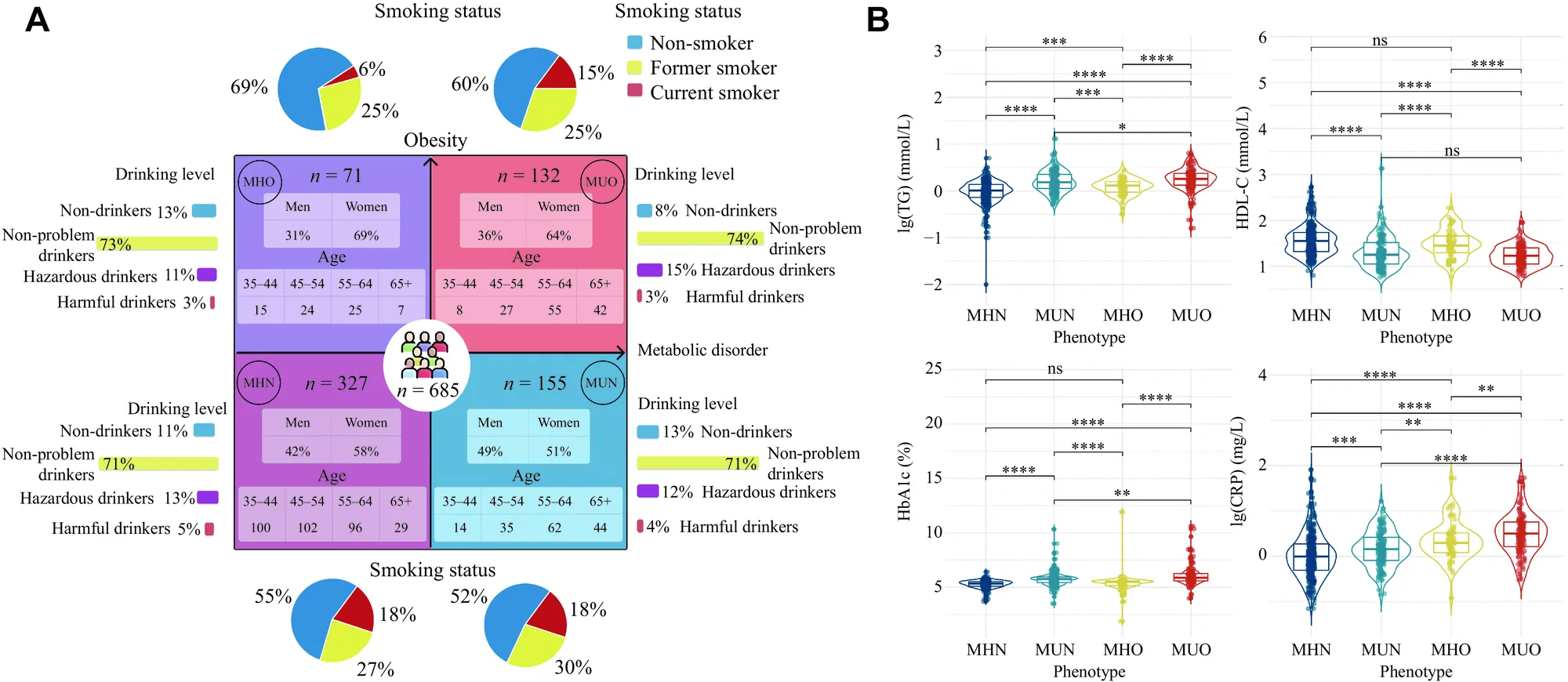
Distribution of study population by phenotypes, sex, age, selected lifestyle variables, and biomarker levels. A: Distribution of the participants. The vertical axis indicates the development of obesity, and the horizontal axis indicates the development of metabolic syndrome (American Heart Association/National Heart, Lung, and Blood Institute, 2009). In each of the colored sectors, a small circle indicates the phenotypes. Each sector indicates the age and sex distribution of patients within that phenotype. The side distributions indicate the drinking level, and the pie charts indicate the current smoking status. B: Violin plots show the results of the Wilcoxon rank-sum test with Benjamini–Hochberg (BH) correction for multiple testing, comparing biomarker levels across phenotypes. The biomarkers shown are: logarithm of C-reactive protein (CRP), logarithm of triglycerides (TG), high-density lipoprotein (HDL-C), and glycated hemoglobin (HbA1c). ^*^P ≤ 0.05, ^**^P ≤ 0.01, ^***^P ≤ 0.001, and ^****^P ≤ 0.0001. Abbreviations: MHN, metabolically healthy non-obese; MHO, metabolically healthy obese; MUN, metabolically unhealthy non-obese; MUO, metabolically unhealthy obese; ns, not significant.

### Characteristics associated with the taxonomic and pathway composition of fecal microbiota

According to the PERMANOVA test with Bray–Curtis distance metric, phenotype, batch, sex, age, education, physical activity, ISCO, diet quality, smoking status, drinking level, cancer, and kidney disease jointly explained approximately 7% of the microbiota taxonomic composition (***Fig. 2***). Depending on the level of agglomeration, the significance of the feature and its contribution to microbiota variation differed. For both agglomeration levels, phenotype was a statistically significant covariate. Other characteristics that significantly contributed to microbiota composition at the genus and ASV levels (*R*^2^ > 0.0055 in the PERMANOVA test, ***Fig. 2***) included sex, age, drinking level, smoking status, ISCO group, and technical batch.

**Figure 2:**
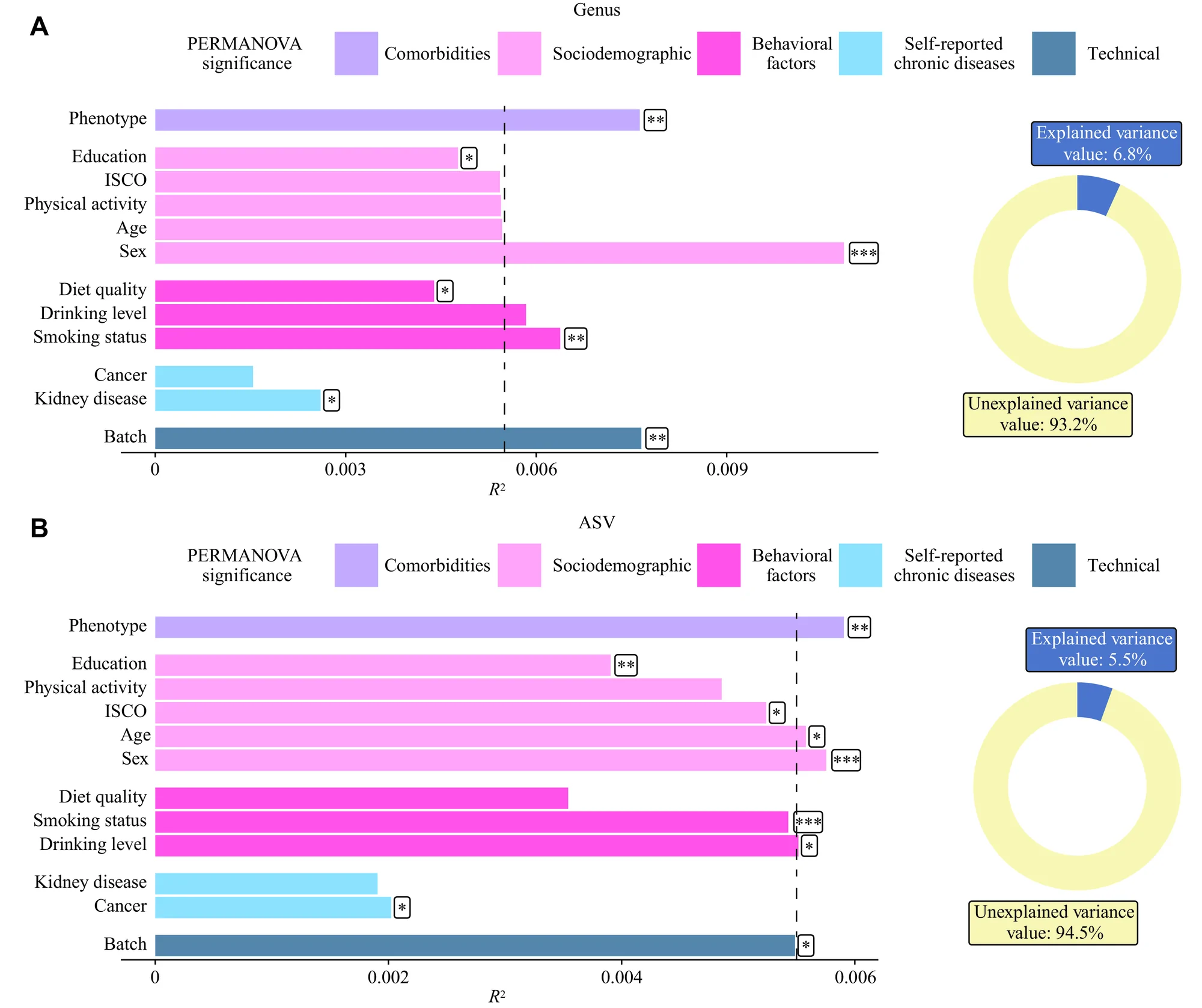
The variance in microbiota composition explained by participants' characteristics. Asterisks indicate the level of significance with the following thresholds: ^*^P ≤ 0.05, ^**^P ≤ 0.01, and ^***^P ≤ 0.001. The agglomeration was performed at the genus (A) and amplicon sequence variant (ASV) (B) levels, with prevalence filtering at 0.05 and detection filtering at 10. The figure shows a permutational multivariate analysis of variance (PERMANOVA) test using Bray–Curtis distances. Statistical significance of group differences was assessed using the adonis2 function (vegan package).

Correspondingly, variation in functional pathway profiles was primarily driven by phenotype, age, sex, ISCO group, and technical batch, together explaining approximately 6% of the functional pathway profiles (***Supplementary Fig. 3***).

### Beta- and alpha-diversity

β-Diversity analysis revealed statistically significant differences in microbiota composition between phenotypes and batches at the ASV level, but not at the genus level. Specifically, phenotypes MHN and MUO, as well as MUN and MUO, exhibited distinct microbial community structures (***Fig. 3***). Similarly, significant compositional differences were observed between batches B1 and B3, as well as B1 and B6 (***Supplementary Fig. 4***). There were no notable differences in α-diversity indices between the phenotypes (***Fig. 4***). At the functional level, statistically significant differences were also observed in β-diversity. Cluster centroid comparisons revealed significant distinctions between MHN and MHO, MHN and MUN, and MHN and MUO (***Fig. 5A***).

**Figure 3:**
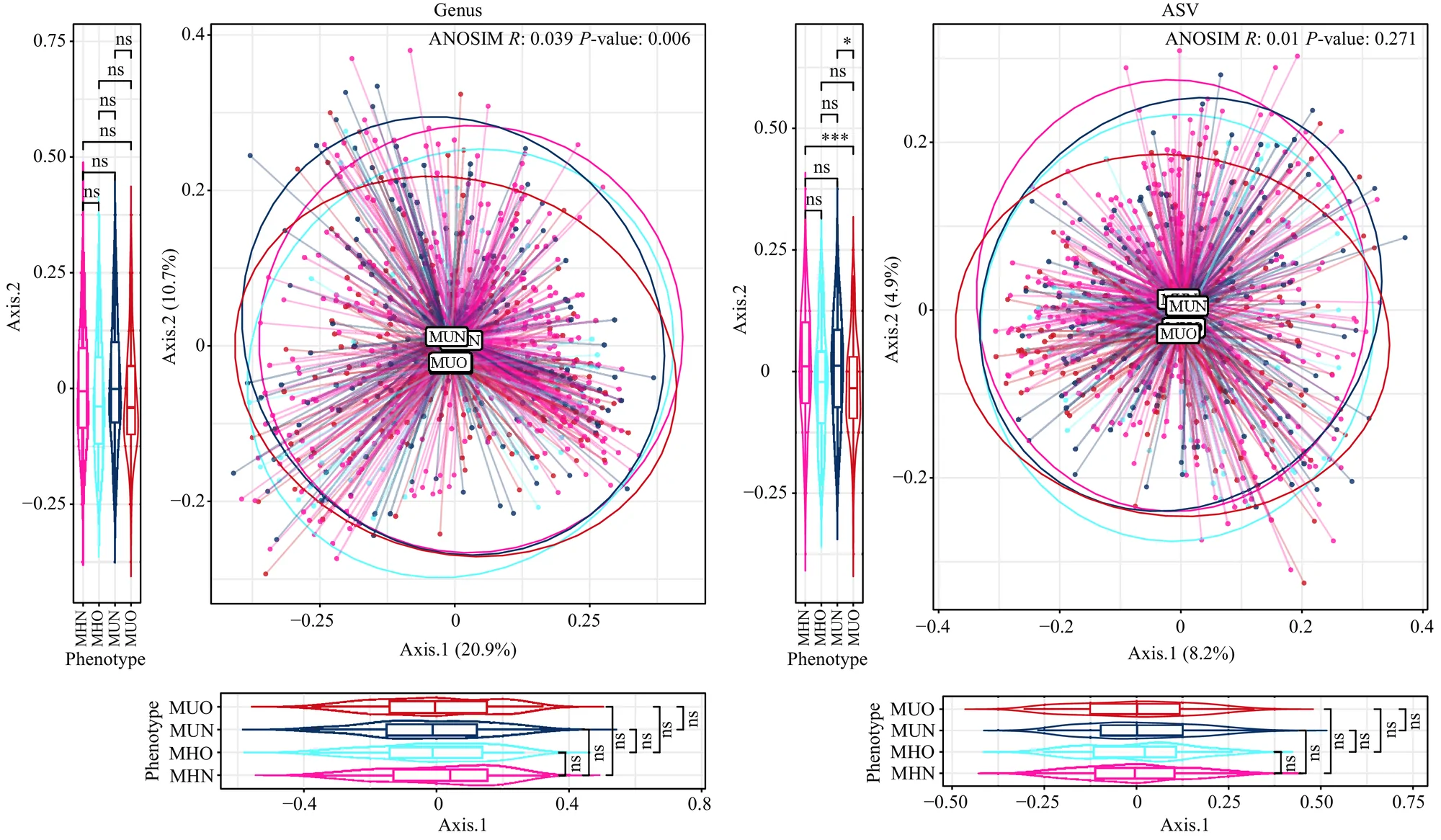
Principal coordinates analysis (PCoA) considering inter-sample Bray–Curtis distance based on the relative abundance of genomes at the genus and amplicon sequence variant (ASV) levels. The violin plot shows the first two components distributed among the phenotypes. A nonparametric, two-sided Wilcoxon rank-sum test with the Benjamini–Hochberg procedure was used for testing the violin plot distributions. Samples were agglomerated and sorted by a prevalence of 0.05. ^*^P ≤ 0.05 and ^***^P ≤ 0.001. Abbreviations: ANOSIM, analysis of similarities; MHN, metabolically healthy non-obese; MHO, metabolically healthy obese; MUN, metabolically unhealthy non-obese; MUO, metabolically unhealthy obese; ns, not significant.

**Figure 4:**
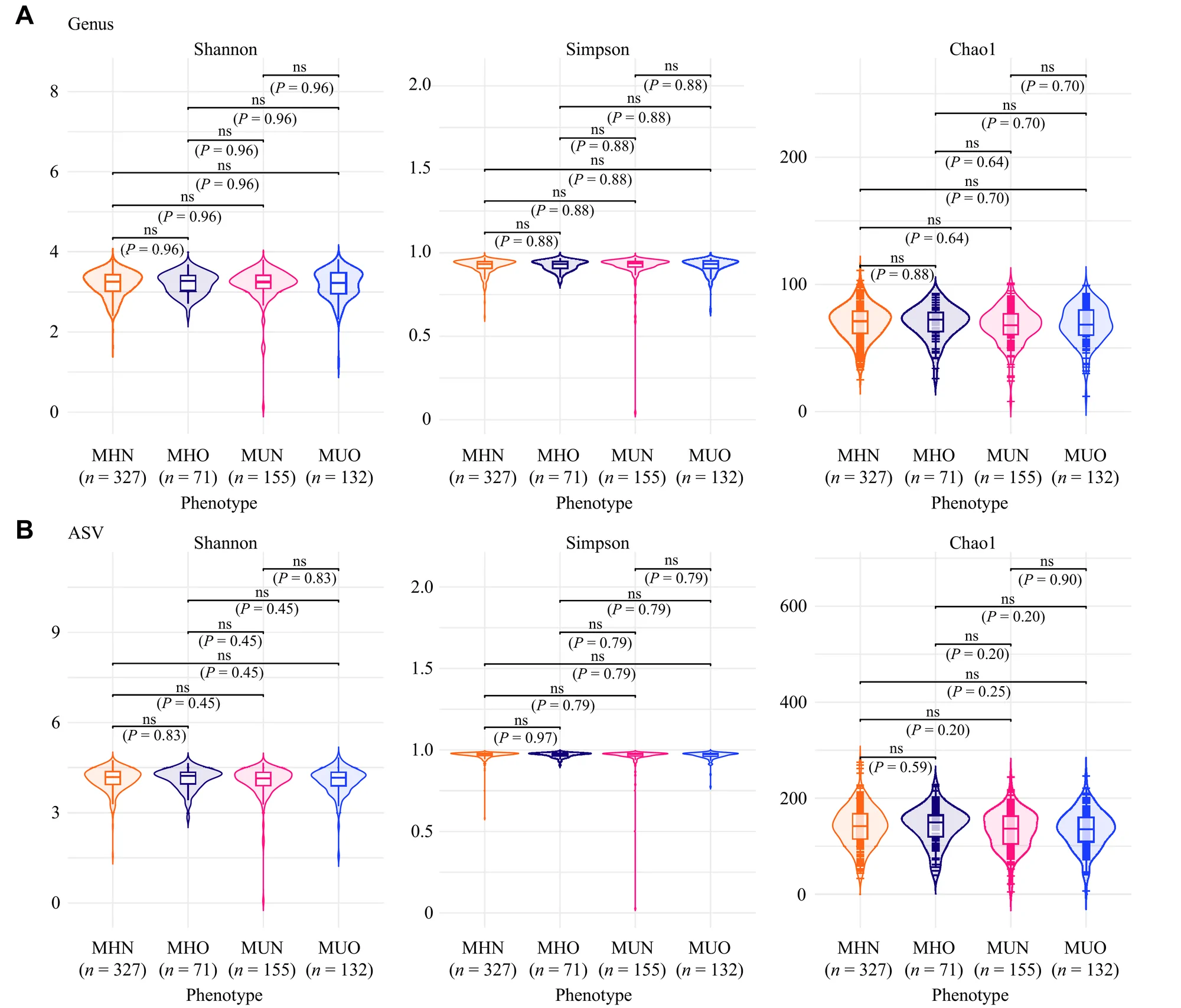
Alpha-diversity analysis. Shannon, Simpson, and Chao1 indices were used. Violin plots illustrate the α-diversity index in the microbiota of samples from 685 participants across the MHN (*n* = 327), MUN (*n* = 155), MHO (*n* = 71), and MUO (*n* = 132) phenotypes. A nonparametric, two-sided Wilcoxon rank-sum test with the Benjamini–Hochberg procedure was used for testing the violin plot distributions. Samples were agglomerated to genus (A) and amplicon sequence variant (ASV) (B) levels and sorted by a prevalence of 0.05. Abbreviations: MHN, metabolically healthy non-obese; MHO, metabolically healthy obese; MUN, metabolically unhealthy non-obese; MUO, metabolically unhealthy obese; ns, not significant.

**Figure 5:**
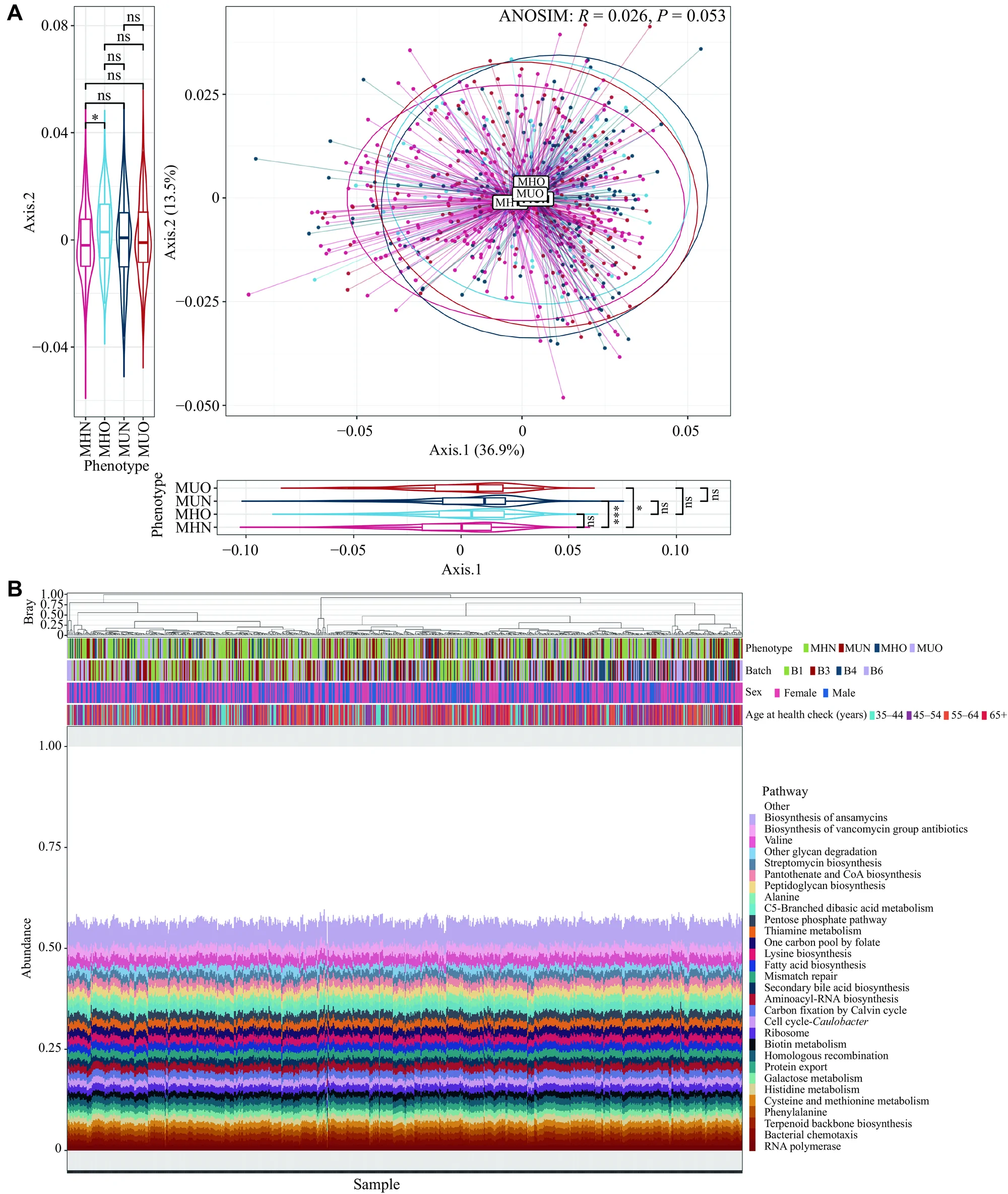
Distribution of microbial pathways in the microbiota. A: Principal coordinates analysis (PCoA) considering inter-sample Bray–Curtis distance based on the relative abundance of KEGG Orthology pathways. The violin plot shows the first two components distributed among the phenotypes. A nonparametric, two-sided Wilcoxon rank-sum test with the Benjamini–Hochberg procedure was used for testing the violin-plot distributions. ^*^P ≤ 0.05 and ^***^P ≤ 0.001. Samples were agglomerated and sorted by a prevalence of 0.05. B: Stacked bar plot with hierarchical clustering using Bray–Curtis distance on the pathway matrix. Each vertical bar on the x-axis represents a single sample, while the y-axis indicates the relative abundance of the 30 most dominant pathways within that sample. For each sample, its group membership is plotted below the dendrogram: phenotype, sex, batch, and age. Abbreviations: MHN, metabolically healthy non-obese; MHO, metabolically healthy obese; MUN, metabolically unhealthy non-obese; MUO, metabolically unhealthy obese; ns, not significant.

### Relative abundance patterns

To compare bacterial community structures across samples, we performed hierarchical clustering based on Bray–Curtis dissimilarities. ***Fig. 6*** indicates the presence of three to four primary clusters, which can be identified by the branching of the tree at approximately 0.8 on the Bray scale. In certain clusters, *Bacteroides* clearly predominates, while in others, *Prevotella_9* is more abundant; in yet others, no dominant taxon is observed. Notably, complete dominance of a single phenotype, batch, sex, or age group within a distinct cluster was not observed; rather, each cluster contains a different feature distribution, though their proportions may vary. Unlike the taxonomic level, microbial pathways demonstrated greater stability across the population (***Fig. 5B***).

**Figure 6:**
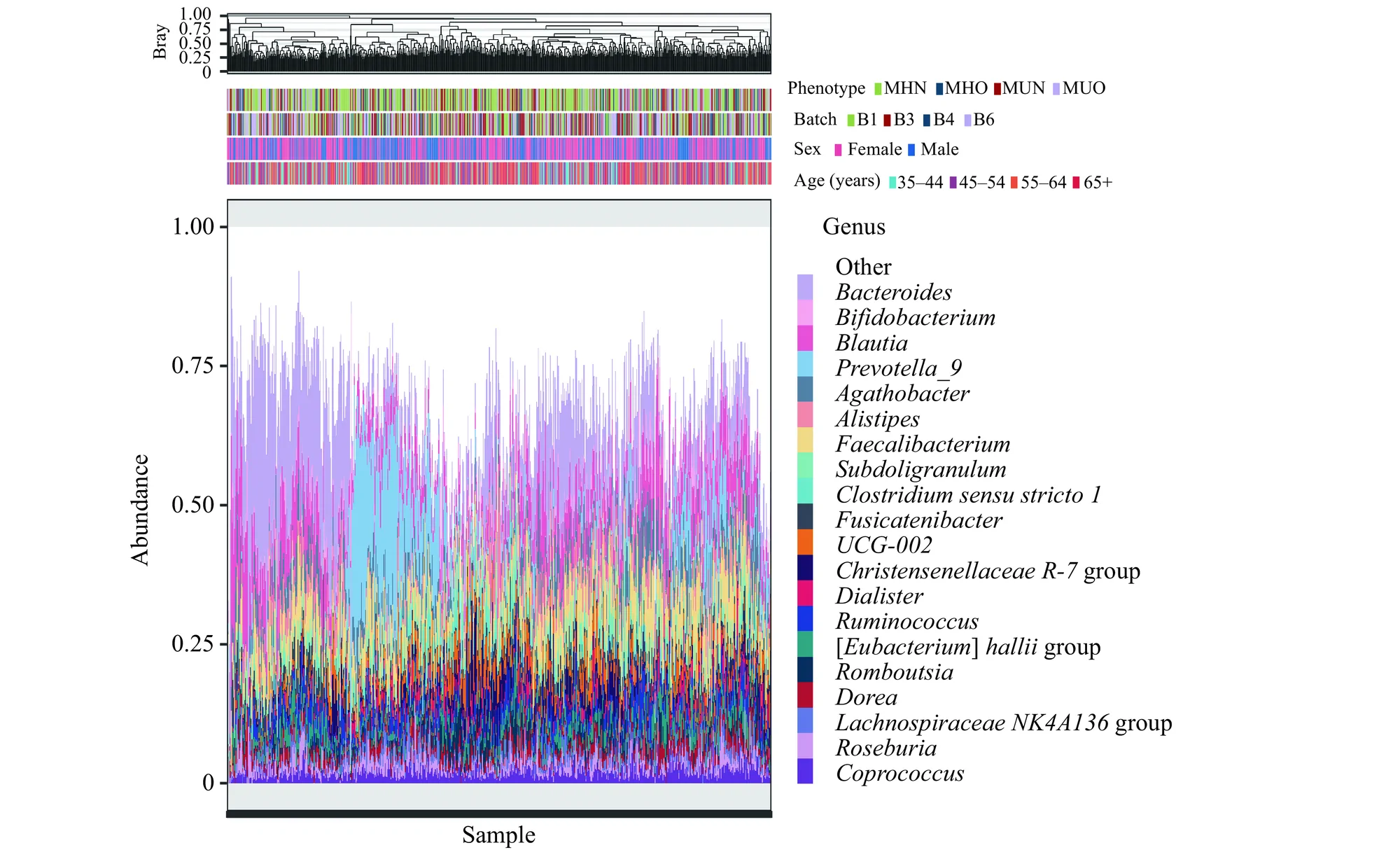
Stacked bar plot with hierarchical clustering using Bray–Curtis distance. Each vertical bar on the x-axis represents a single sample, while the y-axis indicates the relative abundance of the 20 most dominant bacterial genera within that sample. Colors within each bar correspond to different bacterial genera, as detailed in the legend on the right. For each sample, its group membership is plotted below the dendrogram: phenotype, sex, batch, and age. Abbreviations: MHN, metabolically healthy non-obese; MHO, metabolically healthy obese; MUN, metabolically unhealthy non-obese; MUO, metabolically unhealthy obese.

### Microbial enterotypes

Four DMM microbial clusters (enterotypes) were identified based on one of the lowest Laplace approximation scores (***Fig. 7A***). To explore the component composition, we used the Dirichlet parameter vector obtained by fitting a single mixture to the dataset as a reference. The total sum of the posterior mean absolute differences from this reference for the four components was 144%, indicating substantial differences in community structures between each component and the reference (***Supplementary Table 2***). This quantity varies between 0 and 200% for metacommunities that are identical and completely dissimilar to the reference, respectively. We found 10 genera shown in ***Fig. 7B*** accounted for over 48.4% of the observed differences. In particular, *Bacteroides* contributed approximately 15% of this variance. This genus was significantly overrepresented in the first enterotype, accounting for nearly 16.2% of this community, and in the fourth enterotype, where it accounted for approximately 15.5%. In contrast, its representation in the second and third enterotypes was significantly lower, at around 4.4% and 5.8%, respectively.

**Figure 7:**
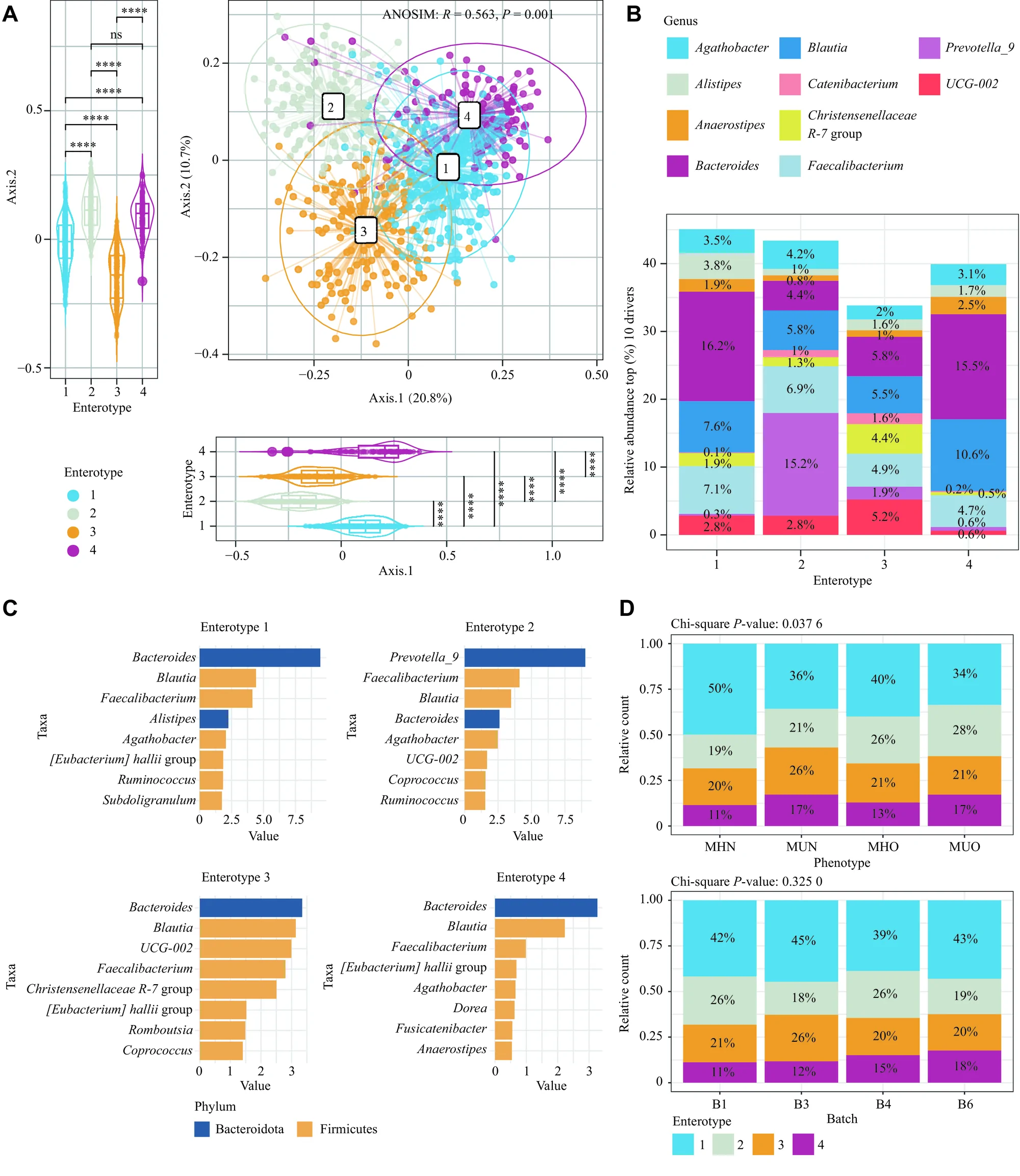
Distribution of taxa by enterotypes and relationship with main metadata. A: Principal coordinates analysis (PCoA) considering inter-sample Bray–Curtis distance based on the relative abundance of genomes *per* the Dirichlet multinomial mixtures (DMM) cluster. A nonparametric two-sided Wilcoxon rank-sum test with the Benjamini–Hochberg procedure was used for testing the violin-plot distributions. ^****^P ≤ 0.0001. Samples were agglomerated to the genus level and sorted by a prevalence of 0.05 and a detection of 1. B: Bar plot showing the relative abundance of the 10 most dominant bacterial genera per DMM cluster. C: Bar plots showing the contribution of each taxonomic group to each enterotype. D: Percentages of the four enterotypes in each phenotype and batch. The association between phenotypes and enterotypes was assessed using the chi-square test (*P* = 0.038). The association between batch groups and community types was assessed using the chi-square test (*P* = 0.325). Abbreviations: MHN, metabolically healthy non-obese; MHO, metabolically healthy obese; MUN, metabolically unhealthy non-obese; MUO, metabolically unhealthy obese; ns, not significant.

Additionally, *Prevotella* accounted for 12.0% of the observed differences between enterotypes and was predominantly represented in the second enterotype, where it comprised 15.2%. *Blautia* contributed 4.0% of the variance between enterotypes and was overrepresented in the fourth enterotype. Taxa defining key features in the microbial community are shown in ***Fig. 7C***. In the first enterotype, *Bacteroides* served as the dominant microbial driver. The second enterotype was characterized by a predominance of *Prevotella*. The fourth enterotype showed a combination of *Bacteroides* and *Blautia* as dominant genera, while the third enterotype showed a balanced representation of *Bacteroides*, *Blautia*, *UCG-002*, and *Faecalibacterium*.

The discovered enterotypes were significantly associated with the studied phenotypes (*P* = 0.038). The first enterotype was predominant in MHN ("healthy" phenotype), accounting for more than half of its composition and exceeding its representation in the "unhealthy" phenotypes (***Fig. 7D***). The second enterotype, dominated by *Prevotella*, accounted for 28% of MUO and 19% of MHN.

Although PERMANOVA showed an association between taxonomic composition and batch, there were no significant differences in enterotype clusters by batch, indicating that these clusters were not driven by this technical factor.

### Phenotype-associated taxonomic signatures

Differential abundance analysis was performed at both the genus and ASV levels (***Fig. 8***). Applying a consensus approach to identify statistically significant taxa, we focused exclusively on comparisons between the MHN and MUO groups.

**Figure 8:**
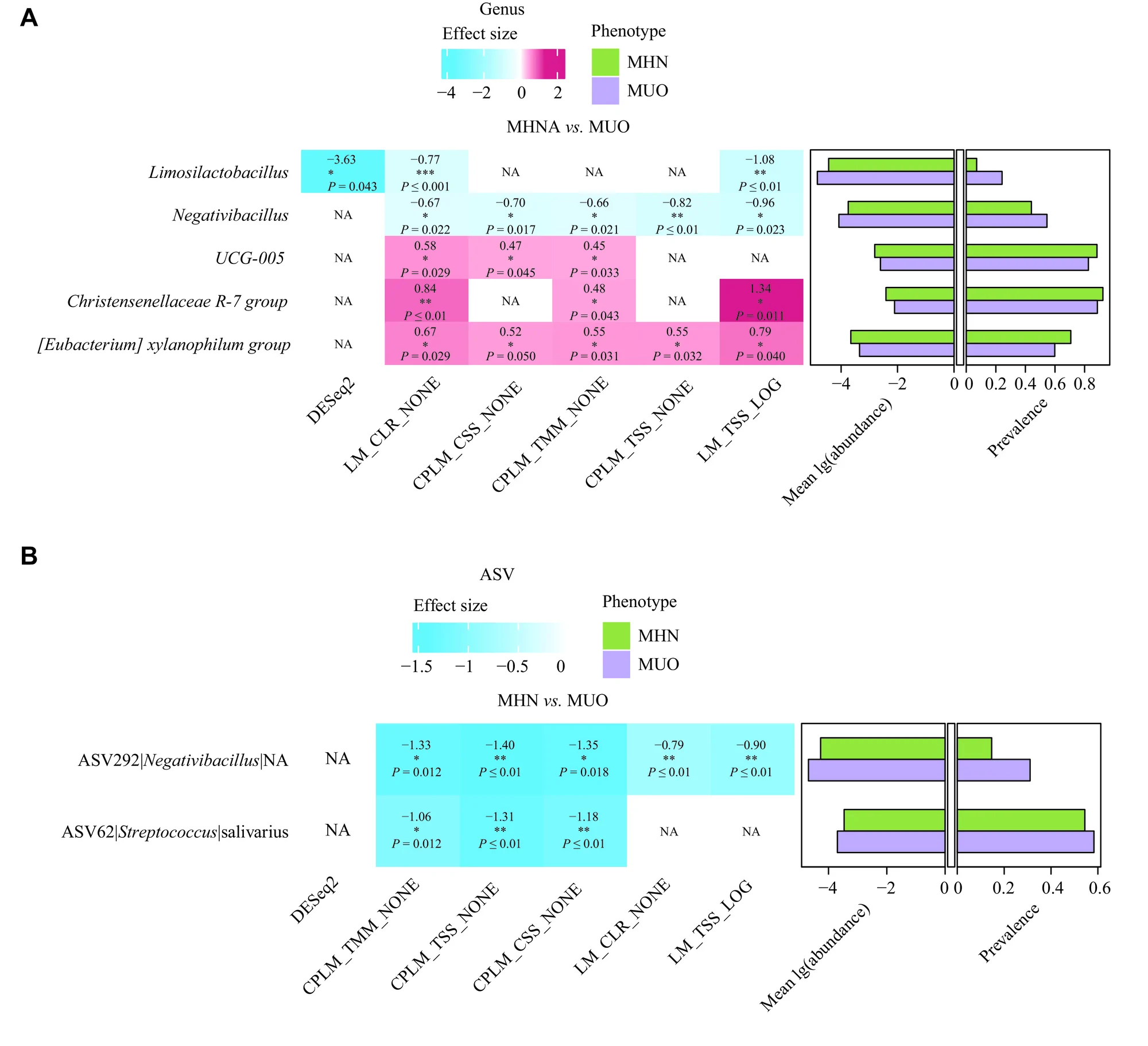
Association testing of differentially abundant taxa. Bacterial taxa at the genus (A) and amplicon sequence variant (ASV) (B) levels that differ in abundance between participants with different phenotypes. Analyses were performed using the Differential Expression analysis for Sequencing data (version 2) (DESeq2) and Microbiome Multivariable Association with Linear Models (MaAsLin 2.0) methods. ^*^P ≤ 0.05, ^**^P ≤ 0.01, and ^***^P ≤ 0.001. The Benjamini–Hochberg procedure was used to correct for multiple comparisons. The mean value of the logarithmic abundance within the group and the prevalence in the comparison group are presented for each taxon. Inside each cell in the heatmap are the effect size, asterisk, and *P*_adjusted_. Abbreviations: MHN, metabolically healthy non-obese; MHO, metabolically healthy obese; MUN, metabolically unhealthy non-obese; MUO, metabolically unhealthy obese; NA, not applicable.

At the genus level, participants in the MHN group exhibited increased abundances of *Christensenellaceae* *R-7 group* (|mean effect size| = 0.87), *Ruminococcaceae UCG-005* (|mean effect size| = 0.5), and *[Eubacterium] xylanophilum group* (|mean effect size| = 0.62), whereas the MUO group showed higher abundances of *Negativibacillus* (|mean effect size| = 0.76) and *Limosilactobacillus* (|mean effect size| = 1.82) (***Fig. 8А***).

At the ASV level, the MUO group demonstrated significantly higher abundances of *Negativibacillus* (|mean effect size| = 1.15) and *Streptococcus salivarius* (|mean effect size| = 1.2) compared with the MHN group (***Fig. 8B***).

Pathway-level differential analysis, performed on 128 pathways retained after filtering from an initial set of 162, revealed statistically significant differences between the MHN and MHO groups. However, effect sizes were modest (maximum absolute effect size = 0.27), indicating limited biological relevance (***Supplementary Fig. 5***). Therefore, these differences are not discussed further.

Pearson correlations were assessed between key clinical parameters, namely, HDL-C, TG, CRP (mg/L), and HbA1c, and differentially abundant microbial taxa. Significant correlations were identified between the *Christensenellaceae R-7 group* and TG (*r* = −0.15, *P*_adjusted_ = 0.001), *Ruminococcaceae UCG-005* and TG (*r* = −0.13, *P*_adjusted_ = 0.005), *Limosilactobacillus* and TG (*r* = 0.09, *P*_adjusted_ = 0.03), *Limosilactobacillus* and HbA1c (*r* = 0.12, *P*_adjusted_ = 0.008), *Limosilactobacillus* and HDL-C (*r* = −0.11, *P*_adjusted_ = 0.02), as well as the *[Eubacterium] xylanophilum group* and HDL-C (*r* = 0.115, *P*_adjusted_ = 0.013) (***Supplementary Fig. 6***).

### Microbial interaction networks

Network analysis revealed distinctive features in the microbial communities of MHN and MUO (***Fig. 9***). A comparison of other groups can be found in ***Supplementary Fig. 7***. The MHN group was characterized by two dominant hub nodes, *Bacteroides* and *UCG-002*. *Blautia* functioned as a hub node in both the MHO and MUO groups; *Bacteroides* was also a hub in MUO. The two networks exhibited similar values for key topological measures, including modularity (0.60 *vs.* 0.70), clustering coefficient/transitivity (0.33 *vs.* 0.35), and natural connectivity (0.009 *vs.* 0.008). In contrast, a striking difference was observed in edge density (0.020 *vs.* 0.006). MHN's graph had many connections between its nodes, while MUO's graph had a low density, meaning it had relatively few connections.

**Figure 9:**
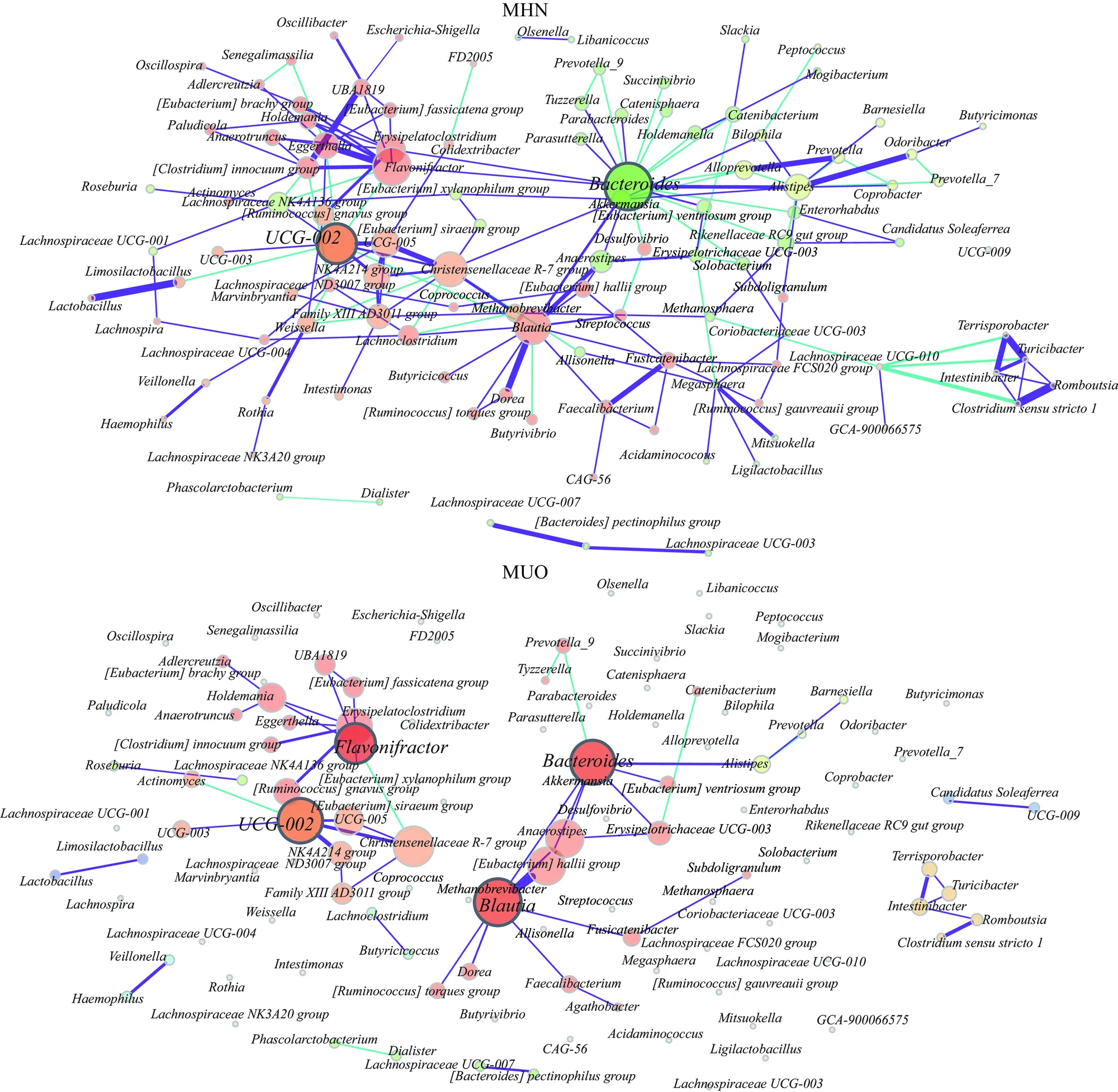
Comparison of the bacterial associations between the MHN (metabolically healthy non-obese) and the MUO (metabolically unhealthy obese). The SParse InversE Covariance Estimation for Ecological Association Inference (SPIEC-EASI) method is employed as a measure of association. The estimated partial correlations are transformed into dissimilarities *via* the "signed" distance metric, and the corresponding similarities are employed as edge weights. Eigenvector centrality is employed to define hubs and scale node sizes. The colors assigned to nodes represent the clusters identified through the application of a greedy modularity optimization algorithm. The coloration of clusters in both networks is consistent if they exhibit at least two shared taxa. Purple edges represent positive estimated associations, while turquoise edges represent negative ones.

## Discussion

To our knowledge, this is the first population study in Northwestern Russia to describe the taxonomic diversity, composition of gut microbiota, and predominant microbial taxa across the four phenotypes, as defined based on the presence or absence of obesity and MetS. There may be regional peculiarities affecting the findings. However, these could not be elucidated because of the lack of comparable population studies of gut microbiota across similarly defined metabolic phenotypes.

Differential abundance analysis is a key methodological approach for identifying differences in taxon abundance between two or more groups. However, this analysis remains a subject of active debate, as the choice of method significantly impacts the reproducibility and interpretation of results, as demonstrated by Nearing *et al*^[27]^. For this reason, we used multiple models to increase the robustness of the results.

In the current study, we employed DESeq2 with the parameter sfType = "poscounts" rather than the default settings. As noted in the function documentation, the "poscounts" estimator provides a robust alternative for datasets containing zeros, which is a common challenge in microbiome data. Specifically, it addresses cases where a taxon has zero counts in some samples by calculating a modified geometric mean based only on non-zero values. This adjustment prevents undefined ratios (which occur when the geometric mean becomes zero) and was developed to accommodate metagenomic data.

Population stratification is a powerful approach to dissecting complex biological systems in human health. When applied to the gut microbiome, this concept has been examined in terms of different community composition types, known as "enterotypes", a theory that has generated considerable interest and debate.

Early work proposed the existence of three canonical enterotypes (Bacteroides-dominated, Prevotella-dominated, and Ruminococcus-dominated)^[28]^. However, Costea *et al*^[29]^ demonstrated that the number and statistical robustness of enterotypes depend heavily on methodological choices. For instance, obesity-related stratification revealed four enterotypes, underscoring the context-dependence of these classifications^[21]^.

Here, we employed DMM to assess microbiota clustering. Notably, our results align with hierarchical clustering approaches, revealing communities dominated by either *Bacteroides* or *Prevotella*, as well as those lacking a clear dominant taxon. This pattern is consistent with prior enterotype frameworks yet highlights the fluidity of such stratification.

Analysis of β-diversity revealed statistically significant differences in the overall microbiota and pathway composition between individuals without obesity (MHN) and those with MUO, indicating differences in microbial community structure and predicted functional profiles between these metabolic phenotypes. Literature data on β-diversity are controversial. A recent systematic review (40 case-control studies) concluded that half of the studies showed a significant change in β-diversity in patients with obesity and MetS (similar to our MUO group) compared with healthy participants without obesity (similar to MHN), while other studies found no significant differences in β-diversity between these groups^[30]^.

We did not observe significant changes in α-diversity across the phenotypes. An earlier study in the Russian population-based sample reported some conflicting results on α-diversity^[31]^. Participants were divided into three groups: "healthy" individuals with normal body weight (similar to MHN), MHO, and MUO. The authors found no differences using the Shannon index, consistent with our results. However, other measures of phylogenetic diversity, including the Chao1 and operational taxonomic unit (OTU) indices, were significantly higher in the MHO participants compared with both the MUO and the "healthy" participants. This suggests increased α-diversity in MHO individuals^[31]^.

Although the literature typically describes microbiota profiles as being divided into three enterotypes based on the dominant bacterial genus (*Prevotella*, *Bacteroides*, and *Ruminococcus*)^[28]^, our study identified four enterotypes. The MHN showed a predominance of the enterotype characterized by a higher abundance of *Bacteroides* species, which is consistent with earlier findings regarding intestinal microbiota composition in "healthy" participants^[6]^. No significant differences in enterotype distribution were observed among the other phenotypes.

The *Christensenellaceae R-7 group* was differentially abundant in the MHN group. *Christensenellaceae*, a recently described family within the *Firmicutes* phylum, represents a subdominant commensal bacterial group that is emerging as a key player in human health. Alongside *Bifidobacterium* and *Akkermansia*, *Christensenellaceae* is recognized as a health- and longevity-associated taxon^[32]^. *Christensenellaceae* has been demonstrated to be associated with weight reduction, and its relative abundance has been shown to be inversely proportional to BMI^[33]^. Furthermore, *Christensenellaceae* is prevalent in many populations, although the specific human genes responsible for its inheritance are still unknown^[34]^. The same study further demonstrated that *Christensenellaceae* acts as a central hub in microbial co-occurrence networks, exhibiting the highest number of connections with other highly heritable taxa. Consistent with these findings, we observed that this family showed significant positive correlations with beneficial commensals, including *Methanobacteriaceae*. In our dataset, we additionally identified significant positive associations between *Christensenellaceae* and several health-associated genera such as *Akkermansia*, *UCG-002*, and *Ruminococcaceae UCG-005* within the MHN group, along with other potentially important taxa.

Most bacteria differentially abundant between the study groups were low-abundance taxa and thus were not detected by either DMM analysis or hierarchical clustering. As noted earlier, *Ruminococcaceae UCG-005* showed a significant positive correlation with *Christensenellaceae R-7 group* and was differentially abundant in the MHN group. This genus has been functionally linked to acetate metabolism^[35]^, potentially contributing to its beneficial role in metabolic health. Notably, in a study investigating short-chain fatty acid metabolism and preeclampsia development in rat models, the relative abundance of *Ruminococcaceae UCG-005* demonstrated a strong negative correlation with systolic blood pressure^[36]^, further supporting its potential protective metabolic functions. These collective findings position *Ruminococcaceae UCG-005* as a taxon of particular interest in microbiota-host metabolic interactions.

The *Eubacterium xylanophilum group* belongs to the family *Lachnospiraceae*. Its fermentation products are formic, acetic, and butyric acids^[37]^. Investigating the effect of polysaccharide from *Enteromorpha clathrata* (ECP) as a probiotic for obesity, it was found that ECP reshaped the structure of the gut microbiota in diseased mice by increasing the abundance of butyrate-producing bacterium, *Eubacterium xylanophilum*, in the gut^[37]^.

One of the bacteria present in greater numbers in the MUO group was *Streptococcus salivarius*. The higher relative abundance of the *Streptococcus* genus in gut disease cohorts confirms its previously reported correlation with a range of gastrointestinal diseases^[38]^. *Streptococcus salivarius* is a commensal organism that may occasionally cause opportunistic infections in humans^[38]^. *Streptococcus salivarius* is one of the first colonizers of the human oral cavity and gut after birth and therefore may contribute to the establishment of immune homeostasis and regulation of host inflammatory responses^[39]^. It has been reported that *Streptococcus salivarius* may help prevent obesity, particularly when there is excessive sucrose intake. It produces exopolysaccharides, which can then be utilized by other gut bacteria to produce short-chain fatty acids, which are beneficial for energy metabolism, reducing fat storage, and improving insulin sensitivity^[39]^.

*Negativibacillus*, a newly identified genus belonging to the *Ruminococcaceae* family of the human gut microbiota, has been implicated as potentially harmful. Its levels were found to be elevated in a mouse model of obesity-related metabolic syndrome. A high-fat diet led to a significant increase in the abundance of *Negativibacillus* in mice, and similar patterns were observed in men and women with MUO, where its presence correlated with greater intake of ultra-processed foods^[40]^. *Negativibacillus* has also been reported to be increased in nonalcoholic fatty liver disease patients compared with a healthy cohort^[41]^. This bacterium remains poorly studied.

*Limosilactobacillus* is a genus of lactic acid bacteria in the family *Lactobacillaceae*, previously part of the broad genus *Lactobacillus*. Some species have been separated into the genus *Limosilactobacillus*, which includes species such as *Limosilactobacillus reuteri*, *Limosilactobacillus fermentum*, *Limosilactobacillus vaginalis*, and others. *Limosilactobacillus fermentum* and *Limosilactobacillus reuteri* are frequently found in the gut and are used as probiotics^[42]^. The anti-obesity action of caffeoylquinic acid through the regulation of thermogenesis was demonstrated, with a functional axis among *L. reuteri*, propionate, and beige fat tissue^[43]^.

In the current study, *Bacteroides* was the predominant hub in the fecal microbiota of MHN, which is consistent with earlier findings of *Bacteroides* associated with lower body weight (similar to MHN)^[44]^. It was also a major hub in MUO, similar to the findings of Tseng *et al*^[34]^. The presence of *Bacteroides* as a hub node in both MHN and MUO suggests a central role in maintaining microbial community structure across metabolic statuses. *Blautia* also emerged as a hub node in both MHO and MUO, which is in line with previous reports of its higher abundance in individuals with obesity (*n* = 1001), highlighting its strong association with visceral fat^[45]^. *Blautia* is known as an acetic acid producer in the intestine, which modulates insulin signaling and fat accumulation in adipocytes by activating specific receptors. This affects the metabolism of unbound lipids and glucose in other tissues, leading to alleviation of obesity-related diseases^[46]^. However, some studies report reduced *Blautia* levels in obese individuals and negative correlations with fasting glucose and HbA1c^[47]^.

Comparative network analysis revealed that obesity-inclusive phenotypes, regardless of the presence of MetS, showed a more disaggregated fecal microbiota structure with a reduced association between different microbial clusters compared with the "healthy" phenotype. This suggests reduced microbial connectivity in obesity-associated phenotypes compared with the MHN phenotype, although the biological and functional implications of these network differences require further investigation.

One limitation of the study is its reliance on 16S rRNA sequencing, which offers lower resolution compared with shotgun metagenomics. This limitation potentially hinders the differentiation of closely related species or strains due to the shorter length of the sequenced regions. Moreover, functional predictions were inferred using PICRUSt2 rather than direct shotgun sequencing, which may underestimate the true functional diversity. Additionally, 16S rRNA sequencing in the current study was performed in several batches. Acknowledging this limitation, we took extensive measures to minimize batch-related variability during sample processing, ensuring consistency across all samples. To account for potential batch effects, we included the categorical variable (batch) in our analysis when calculating the differential abundance of taxa.

Another limitation is that the cross-sectional design of the study did not allow for establishing causal relationships between obesity, metabolic disorders, and the intestinal microbial composition. Changes in the intestinal microbiota can both contribute to and result from obesity and metabolic disorders. On the one hand, there is evidence that specific changes in the intestinal microbiota promote weight gain by influencing energy metabolism, fat storage, and inflammation. Experiments in mice have shown that phenotypes can be transferred from obese to normal-weight animals by gut microbiota transplantation, suggesting that the intestinal microbiota can affect weight regulation and influence metabolic health^[48]^. On the other hand, obesity and metabolic disorders can cause shifts in the intestinal microbiota due to changes in diet, immune responses, and metabolic processes. The relationship is bidirectional, with multiple factors at play.

In conclusion, gut microbial composition differed across metabolic phenotypes, with the most notable taxonomic differences observed between MHN and MUO participants. Several taxa associated with metabolic health were enriched in the MHN group, whereas other taxa were more abundant in the MUO group. These findings suggest that distinct gut microbial signatures are associated with obesity and metabolic dysfunction, although the cross-sectional design precludes causal inference.

## Additional information

The online version contains supplementary material available at http://www.jbr-pub.org.cn/article/doi/10.7555/JBR.39.20250258.
