# Supplementary material for: Taxonomic diversity of fecal microbiota associated with different metabolic phenotypes in residents of Arkhangelsk, Northwestern Russia
Source: J Biomed Res. 2026 Sep 28;40(5):536–52. doi: 10.7555/JBR.39.20250258 (PMC13620460; doi:10.7555/JBR.39.20250258)
Supplement: Supplementary file 1 [file jbr-40-5-536-S1.pdf]

Available online at www.jbr-pub.org.cn

Open Access at PubMed Central

JBR

Journal of Biomedical Research, 2026 40(5): 1–10

Supplementary Data

Taxonomic diversity of fecal microbiota associated with different metabolic phenotypes in residents of Arkhangelsk, Northwestern Russia

Anna Postoeva[1,✉], Ekaterina Krieger[2,3], Anna Leontyeva[1], Alexander V. Kudryavtsev[2], Julia Galeeva[4], Dmitry Fedorov[4], Polina Kuzmichenko[4], Elena Ilina[4], Vadim Govorun[4]

[1]*Department of Hospital Therapy and Endocrinology, Northern State Medical University, Arkhangelsk 163000, Russia;*
[2]*International Research Competence Centre, Central Scientific Research Laboratory, Northern State Medical University, Arkhangelsk 163000, Russia;*
[3]*Department of Infectious Diseases, Northern State Medical University, Arkhangelsk 163000, Russia;*
[4]*Research Institute for Systems Biology and Medicine, Department of Mathematical Biology and Bioinformatics, Moscow 117246, Russia.*

✉Corresponding author: Anna Postoeva, E-mail: ann-primak@yandex.ru, ORCID: 0000-0003-3749-0173.

Received: 28 June 2025; Revised: 07 October 2025; Accepted: 16 October 2025; Available online: 21 October 2025; Published date: 28 September 2026

CLC number: R589, Document code: A

The authors reported no conflicts of interest.

This is an open access article under the Creative Commons Attribution (CC BY 4.0) license, which permits others to distribute, remix, adapt and build upon this work, for commercial use, provided the original work is properly cited.

© 2026 by Journal of Biomedical Research.

https://doi.org/10.7555/JBR.39.20250258

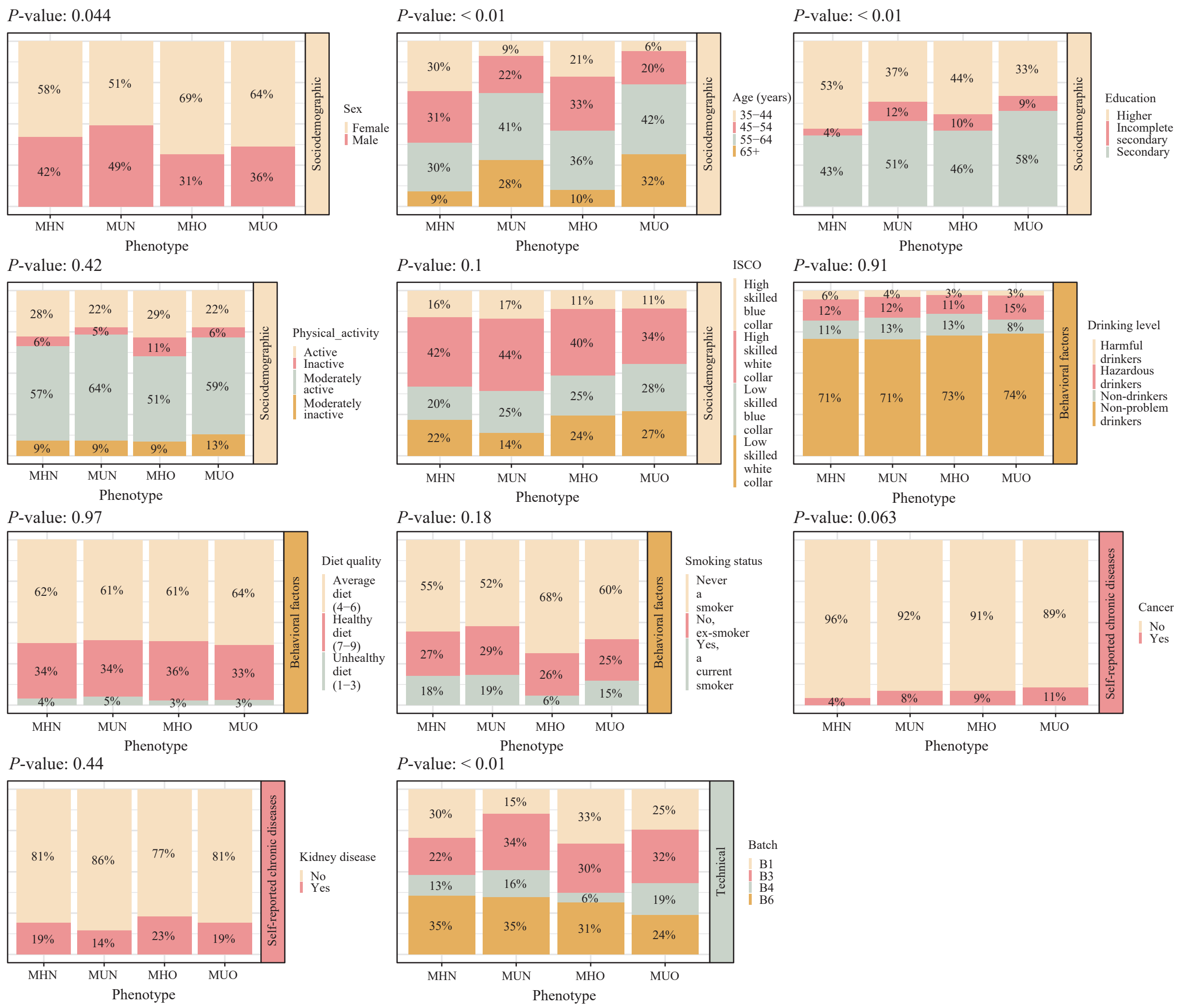

Supplementary Fig. 1 **Relative abundance of phenotypes across various features.** The figure shows the relative representations of different features in each of the phenotypes. A chi-square test was performed for each feature pair to assess the statistical significance of the association between the phenotype and the respective feature. The corresponding *P*-values are indicated in the titles of each subplot.

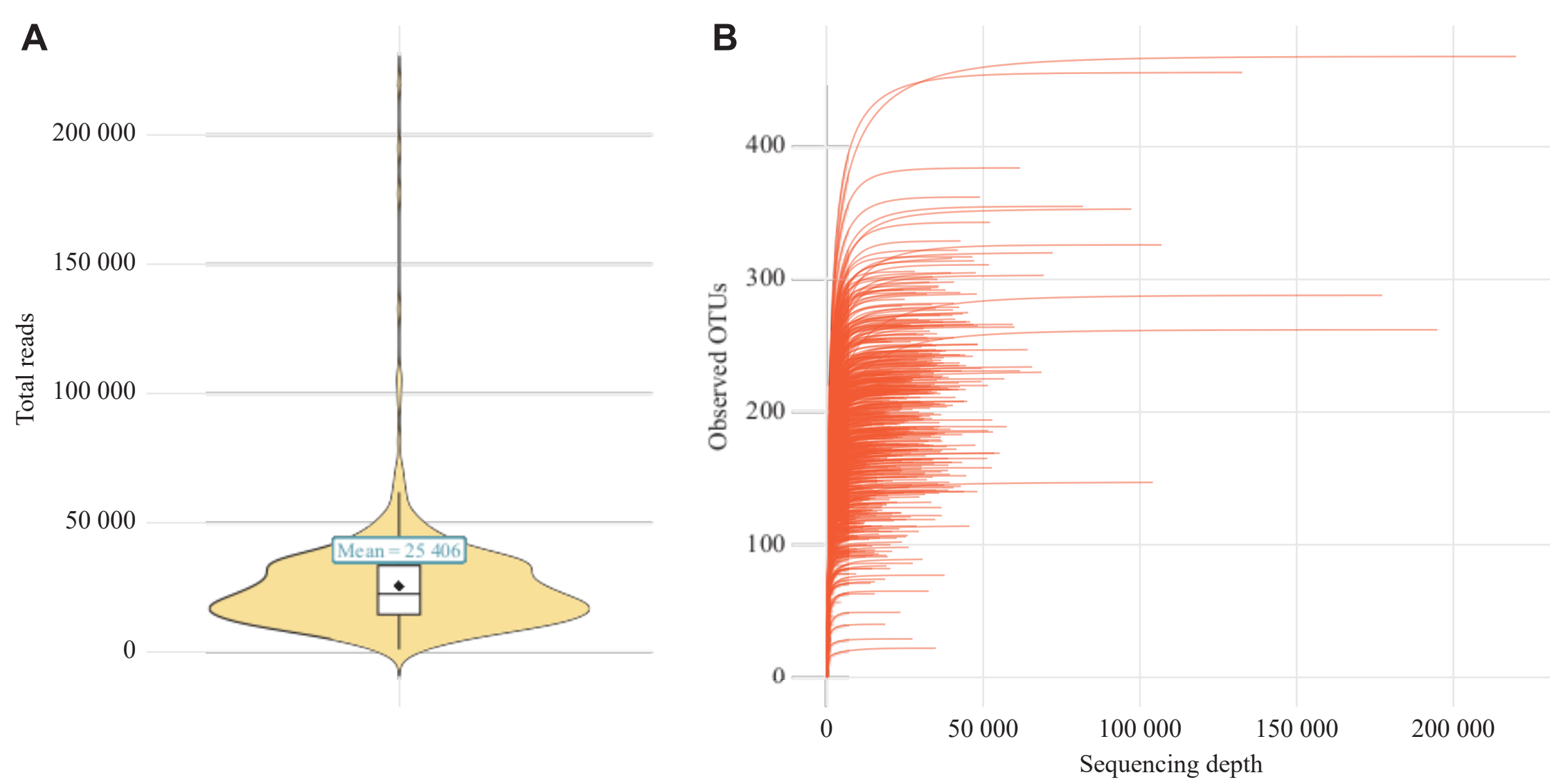

Supplementary Fig. 2 **Sequencing depth and rarefaction analysis of samples.** A: Distribution of total sequencing reads per sample, with the mean number of reads indicated. B: Rarefaction curves for individual samples showing the relationship between sequencing depth and observed operational taxonomic unit (OTU) richness.

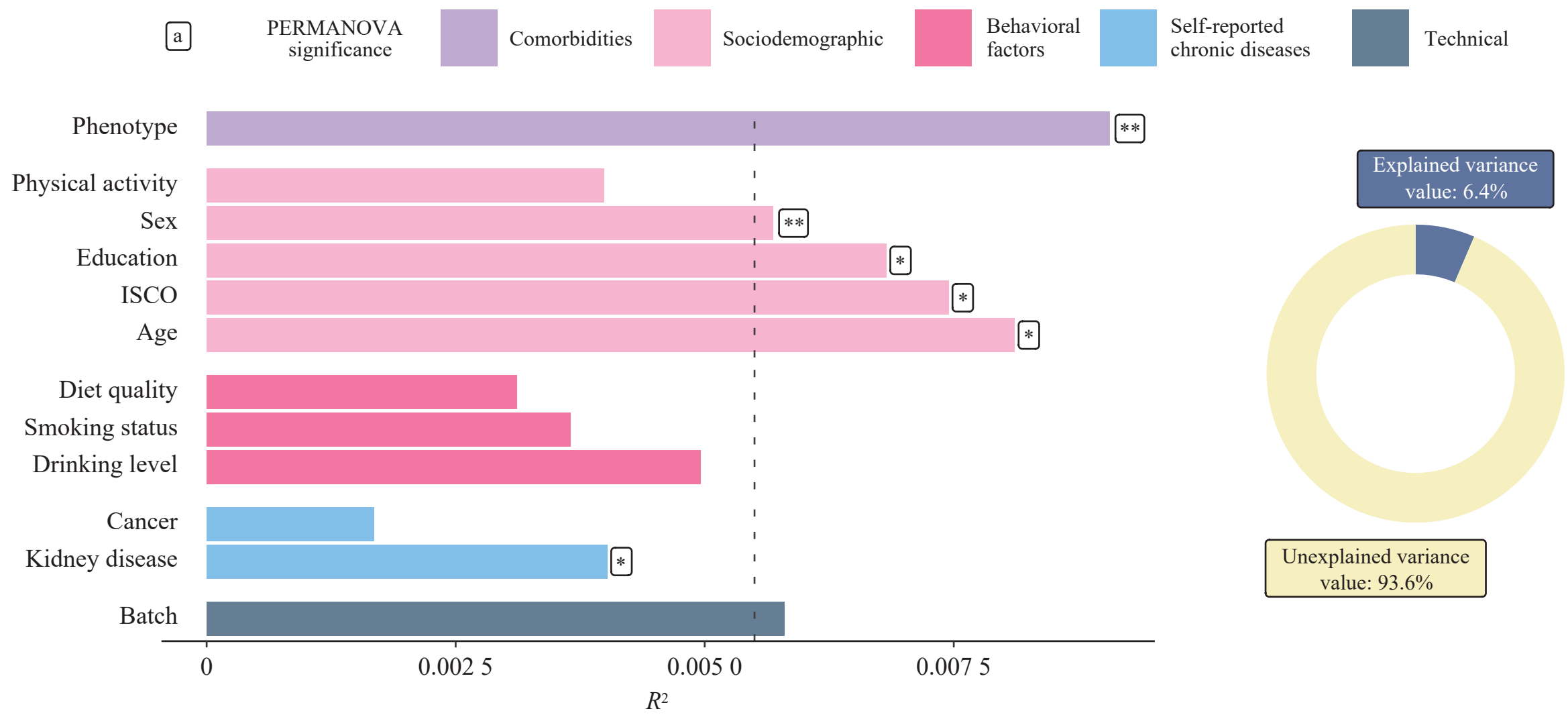

Supplementary Fig. 3 **The variance in microbiota functional composition explained by participants' characteristics.** The variance in microbiota functional composition explained by participants' characteristics. *$P \leqslant 0.05$ and **$P \leqslant 0.01$. The agglomeration was performed at the level of the KO pathway, prevalence filtering at the level of 0.05, and detection filtering at the level of 10. The panel shows permutational multivariate analysis of variance (PERMANOVA) test using Bray–Curtis distances. Statistical significance of group differences was assessed using adonis2 function (vegan package).

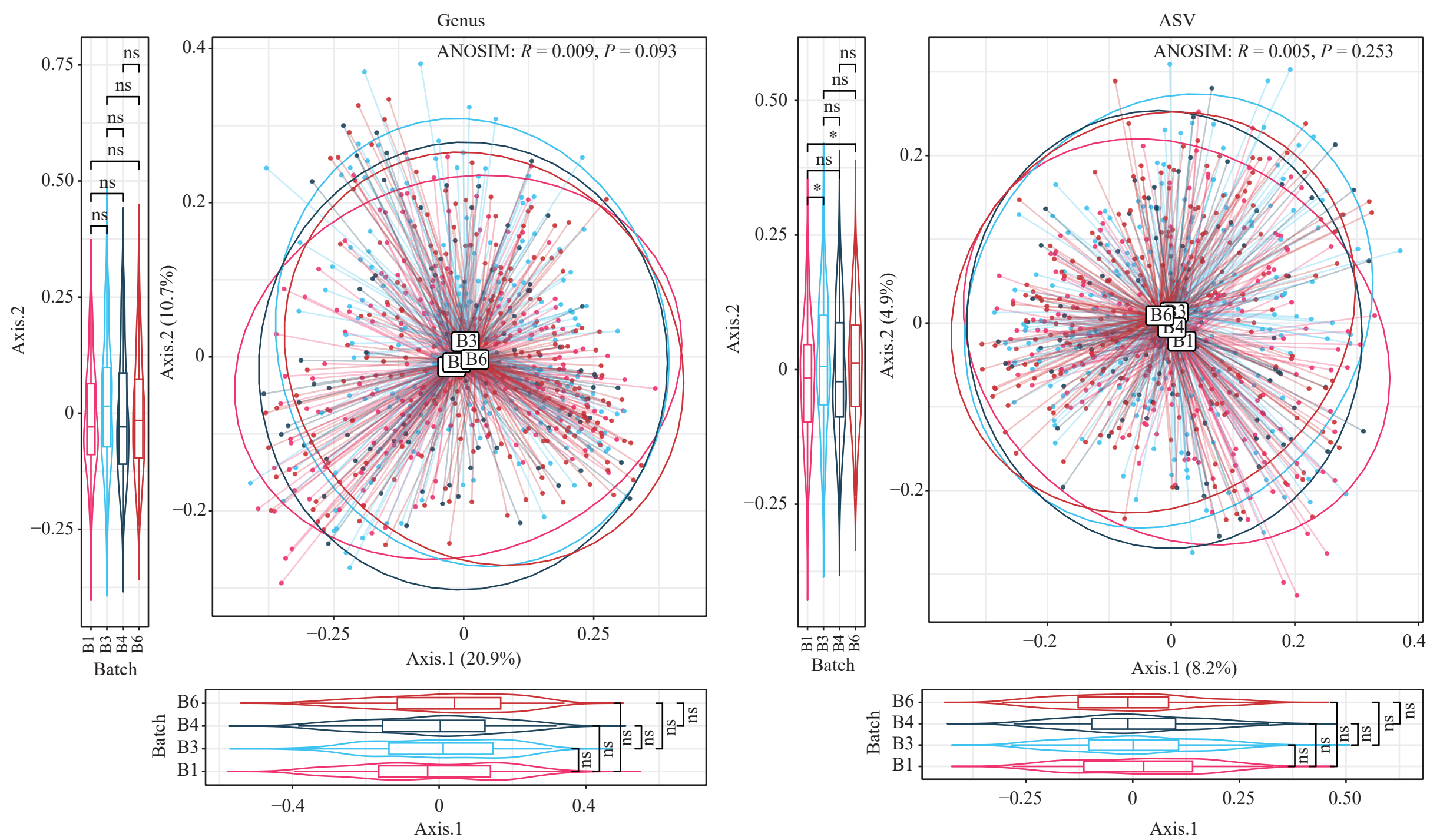

Supplementary Fig. 4 **Principal coordinates analysis (PCoA) considering inter-sample Bray–Curtis distance based on the relative abundance of genomes at genus (A) and amplicon sequence variant (ASV) (B) levels.** The violin plot shows the first two components distributed among the batch. A nonparametric two-sided Wilcoxon rank-sum test with the Benjamini–Hochberg procedure was used for testing the violin-plot distributions. *$P \leqslant 0.05$. Samples were agglomerated and sorted by a prevalence of 0.05. Abbreviation: ns, not significant.

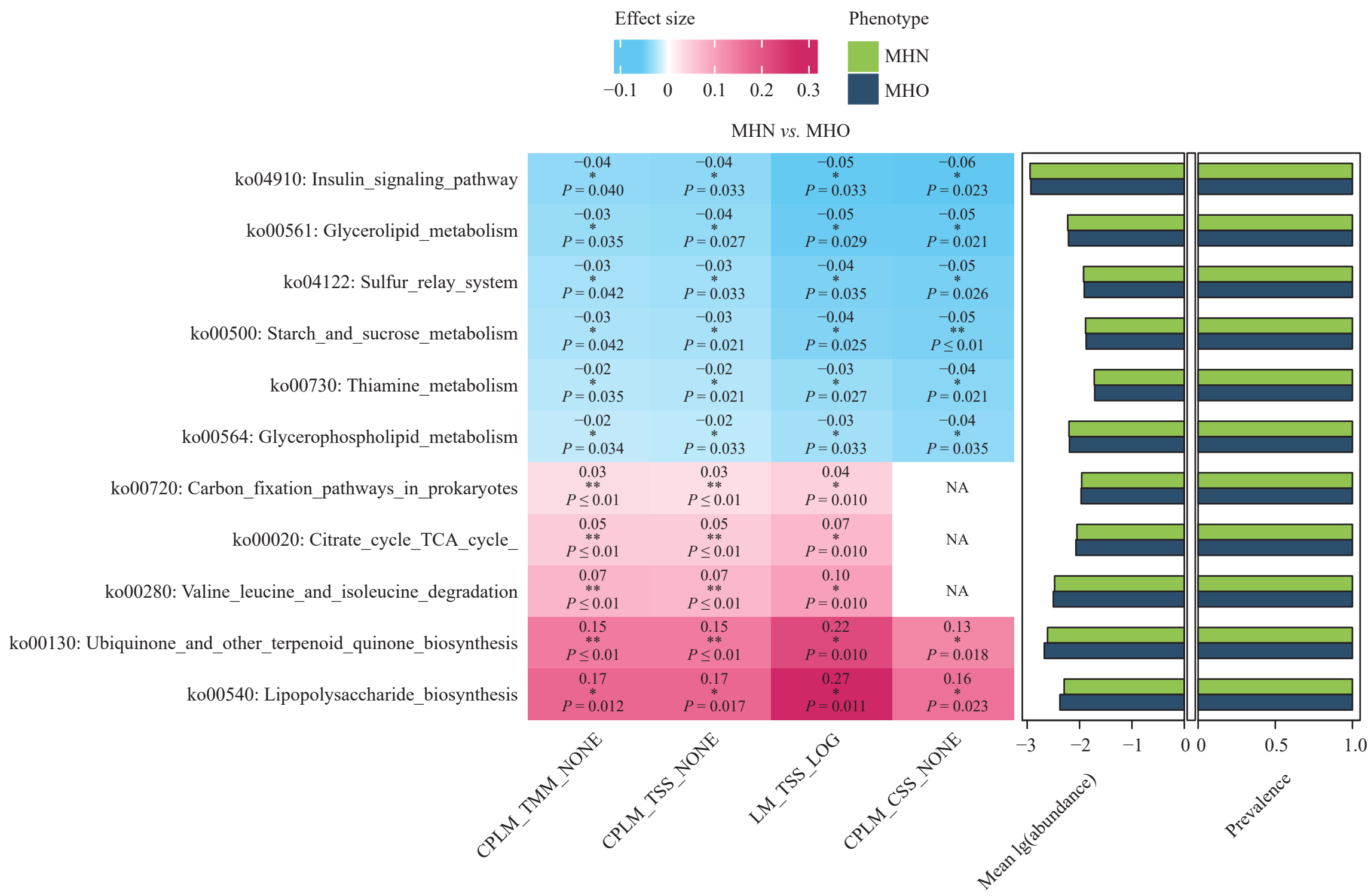

Supplementary Fig. 5 **Association testing of differentially abundant KO pathways.** Analyses were performed using the Differential Expression analysis using Sequencing data (version 2) (DESeq2) and Microbiome Multivariable Association with Linear Models (MaAsLin 2.0) methods. $^{*}P \leqslant 0.05$ and $^{**}P \leqslant 0.01$. The Benjamin-Hochberg procedure was used to correct for multiple comparisons. The mean value of the logarithmic abundance within the group and the prevalence in the comparison group are presented for each taxon. Inside each cell in the heatmap are the effect size, asterisk, and adjusted *P*-values.

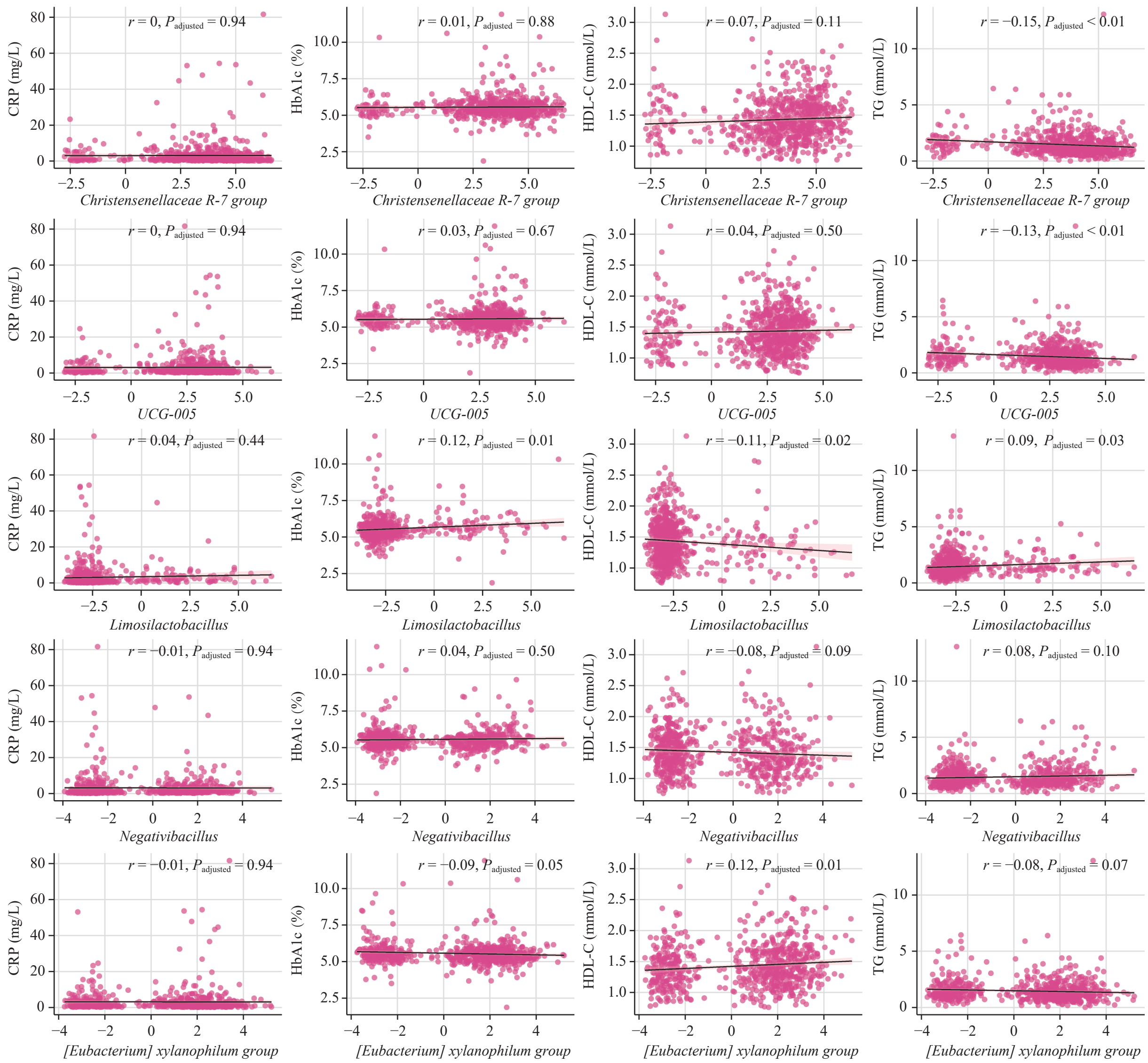

Supplementary Fig. 6 **Correlation analysis of differential taxa and biochemical parameters.** Pearson's correlation analyses between biochemical parameters and centered log ratio (CLR)-transformed abundances of differentially represented taxa were performed using the cor.test function from the stats package (R version 4.2.0). Multiple testing correction was applied to *P*-values using the Benjamini–Hochberg procedure. Abbreviations: CRP, C-reactive protein; HbA1c, glycated hemoglobin; HDL-C, high-density lipoprotein cholesterol; TG, triglycerides.

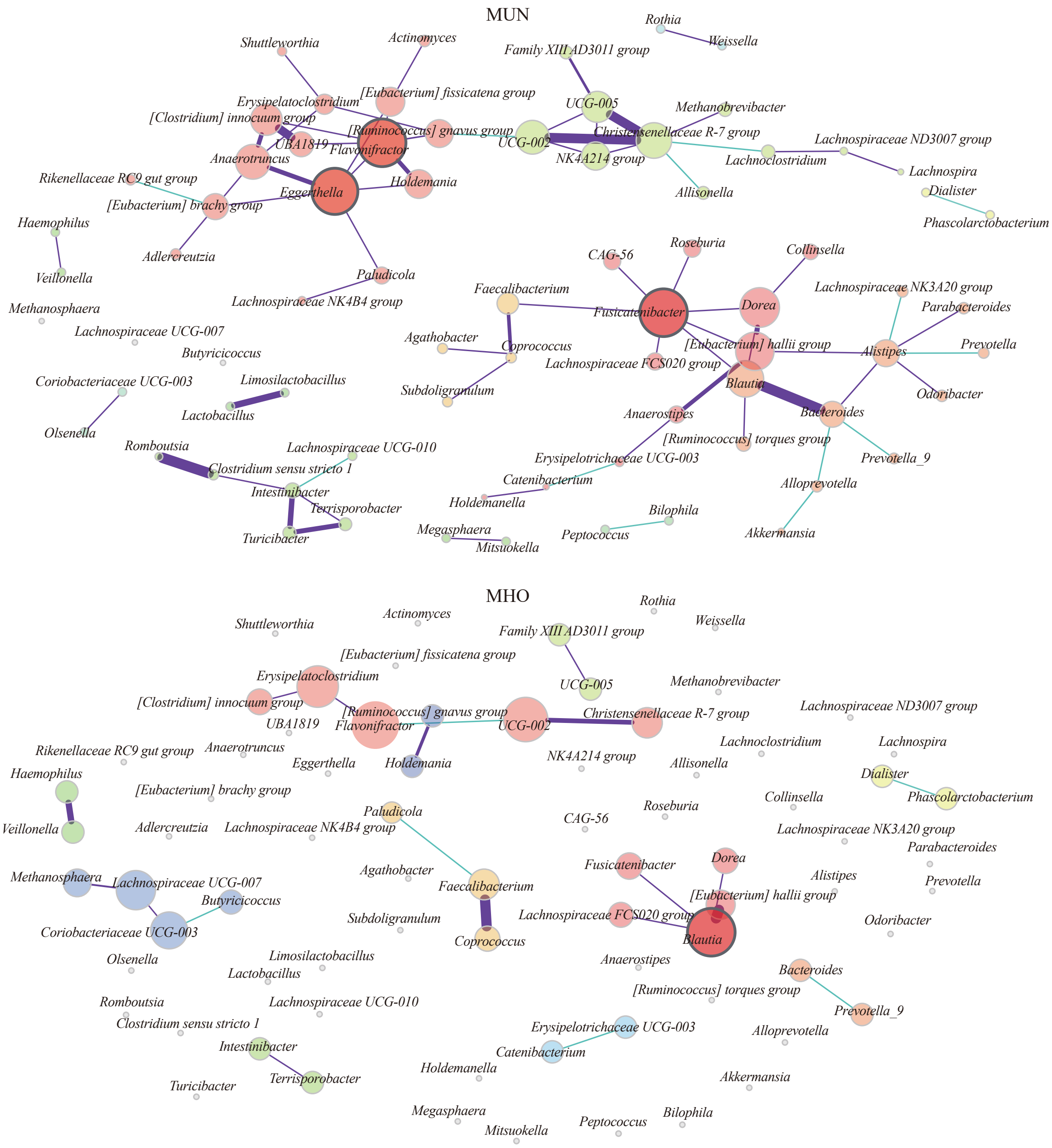

Supplementary Fig. 7 **Comparison of the bacterial associations between the MUN (metabolically unhealthy non-obese) and the MHO (metabolically healthy obese).** The SParse InversE Covariance Estimation for Ecological Association Inference (SPIEC-EASI) method was employed as a measure of association. The estimated partial correlations were transformed into dissimilarities *via* the "signed" distance metric, and the corresponding similarities were employed as edge weights. To define hubs and to scale node sizes, the eigenvector centrality was employed. The colors assigned to nodes represent the clusters identified through the application of a greedy modularity optimization algorithm. The coloration of clusters in both networks is consistent if they exhibit at least two shared taxa. The purple edges represent positive estimated associations, while the turquoise edges represent negative ones.

Supplementary Table 1 **Metadata distribution across samples**

Phenotype	Mean age (years)	Mean triglycerides (mmol/L)	Mean HDL-C (mmol/L)	Mean HbA1c (%)	Mean CRP (mg/L)
MHN	51.72	1.09	1.56	5.29	2.40
MUN	58.91	1.87	1.3	5.78	2.19
MHO	52.84	1.3	1.48	5.38	3.82
MUO	59.51	2.03	1.23	6.08	5.61

Abbreviations: CRP, C-reactive protein; HbA1c, glycated hemoglobin; HDL-C, high-density lipoprotein cholesterol; MHN, metabolically healthy non-obese; MHO, metabolically healthy obese; MUN, metabolically unhealthy non-obese; MUO, metabolically unhealthy obese.

Supplementary Table 2 **Genera frequencies in the enterotypes**

Taxa	Mean	m1	m2	m3	m4	diff	cdiff
Bacteroides	0.098	0.162	0.044	0.058	0.155	0.215	0.148
Prevotella_9	0.010	0.003	0.152	0.019	0.006	0.162	0.261
Blautia	0.072	0.076	0.058	0.055	0.106	0.068	0.308
UCG-002	0.025	0.028	0.028	0.052	0.006	0.052	0.344
Christensenellaceae R-7 group	0.017	0.019	0.013	0.044	0.005	0.045	0.374
Faecalibacterium	0.063	0.071	0.069	0.049	0.047	0.044	0.405
Alistipes	0.022	0.038	0.010	0.016	0.017	0.038	0.432
Anaerostipes	0.016	0.019	0.008	0.010	0.025	0.027	0.450
Agathobacter	0.034	0.035	0.042	0.020	0.031	0.025	0.467
Catenibacterium	0.004	0.001	0.010	0.016	0.002	0.024	0.484
Romboutsia	0.014	0.011	0.011	0.026	0.009	0.023	0.499
Lachnoclostridium	0.012	0.010	0.014	0.006	0.024	0.021	0.514
Holdemanella	0.006	0.002	0.012	0.013	0.002	0.021	0.528
Clostridium sensu stricto 1	0.013	0.011	0.011	0.024	0.008	0.020	0.542
Fusicatenibacter	0.020	0.022	0.014	0.013	0.026	0.020	0.556
Ruminococcus	0.027	0.031	0.026	0.024	0.016	0.020	0.570
Streptococcus	0.009	0.007	0.008	0.004	0.020	0.019	0.583
Subdoligranulum	0.026	0.030	0.021	0.024	0.020	0.018	0.595
UCG-005	0.010	0.009	0.009	0.018	0.003	0.017	0.607
Lachnospiraceae NK4A136 group	0.017	0.016	0.023	0.014	0.009	0.017	0.618
NK4A214 group	0.011	0.010	0.009	0.017	0.004	0.016	0.629
[Ruminococcus] torques group	0.019	0.016	0.015	0.016	0.025	0.015	0.639
Roseburia	0.022	0.020	0.026	0.016	0.018	0.015	0.650
[Eubacterium] hallii group	0.031	0.031	0.023	0.026	0.032	0.015	0.660
Rikenellaceae RC9 gut group	0.003	0.001	0.004	0.011	0.000	0.014	0.669
Butyricicoccus	0.010	0.008	0.008	0.008	0.016	0.013	0.678
Desulfovibrio	0.005	0.002	0.005	0.011	0.002	0.012	0.686
Erysipelotrichaceae UCG-003	0.006	0.010	0.003	0.002	0.007	0.012	0.694
Alloprevotella	0.003	0.001	0.008	0.004	0.001	0.011	0.702
Dorea	0.026	0.022	0.025	0.023	0.030	0.011	0.710
Family XIII AD3011 group	0.006	0.005	0.004	0.011	0.003	0.011	0.718
Bifidobacterium	0.014	0.012	0.009	0.014	0.018	0.011	0.725
Collinsella	0.015	0.012	0.013	0.018	0.013	0.010	0.732
Coprococcus	0.025	0.024	0.026	0.024	0.018	0.010	0.739
Escherichia-Shigella	0.005	0.003	0.003	0.007	0.008	0.009	0.745
[Eubacterium] ventriosum group	0.005	0.006	0.002	0.002	0.007	0.008	0.751
Lachnospira	0.009	0.007	0.011	0.006	0.009	0.008	0.756
Akkermansia	0.005	0.007	0.002	0.006	0.004	0.008	0.761
Methanobrevibacter	0.003	0.002	0.001	0.006	0.000	0.007	0.767
Intestinibacter	0.006	0.004	0.004	0.009	0.007	0.007	0.772
Parabacteroides	0.014	0.013	0.011	0.012	0.013	0.007	0.776
Odoribacter	0.008	0.008	0.005	0.007	0.005	0.007	0.781

Supplementary Table 2 **Genera frequencies in the enterotypes** (continued)

Taxa	Mean	m1	m2	m3	m4	diff	cdiff
[Eubacterium] xylanophilum group	0.005	0.006	0.003	0.003	0.002	0.007	0.785
Barnesiella	0.005	0.006	0.003	0.004	0.004	0.006	0.790
Intestinimonas	0.006	0.004	0.006	0.008	0.004	0.006	0.794
Dialister	0.006	0.004	0.007	0.008	0.008	0.006	0.798
UCG-003	0.006	0.006	0.005	0.006	0.002	0.006	0.803
Marvinbryantia	0.004	0.003	0.003	0.006	0.002	0.006	0.807
Monoglobus	0.010	0.008	0.007	0.008	0.010	0.006	0.811
[Eubacterium] ruminantium group	0.003	0.002	0.004	0.004	0.001	0.006	0.815
Lachnospiraceae UCG-010	0.004	0.004	0.004	0.002	0.007	0.006	0.819
Prevotella	0.002	0.000	0.005	0.003	0.001	0.006	0.823
Solobacterium	0.002	0.001	0.003	0.004	0.001	0.006	0.827
Sutterella	0.006	0.004	0.008	0.005	0.006	0.006	0.831
Flavonifractor	0.001	0.000	0.000	0.000	0.005	0.005	0.834
[Eubacterium] eligens group	0.005	0.003	0.005	0.005	0.001	0.005	0.838
Senegalimassilia	0.004	0.003	0.004	0.006	0.003	0.005	0.842
Incertae Sedis	0.008	0.006	0.005	0.008	0.008	0.005	0.845
Lachnospiraceae ND3007 group	0.009	0.007	0.008	0.006	0.009	0.005	0.848
[Eubacterium] siraeum group	0.005	0.005	0.004	0.005	0.002	0.005	0.852
Slackia	0.004	0.002	0.003	0.005	0.002	0.005	0.855
Enterorhabdus	0.002	0.001	0.002	0.004	0.001	0.005	0.858
Howardella	0.003	0.002	0.003	0.004	0.002	0.005	0.861
Oscillibacter	0.004	0.003	0.002	0.004	0.005	0.004	0.864
Butyricimonas	0.005	0.004	0.004	0.005	0.002	0.004	0.867
[Ruminococcus] gauvreauii group	0.009	0.007	0.009	0.008	0.009	0.004	0.870
Butyrivibrio	0.002	0.001	0.002	0.003	0.000	0.004	0.873
CAG-56	0.004	0.004	0.003	0.002	0.005	0.004	0.876
Tyzzerella	0.001	0.002	0.000	0.000	0.003	0.004	0.879
Paraprevotella	0.003	0.002	0.004	0.003	0.002	0.004	0.882
[Ruminococcus] gnavus group	0.001	0.000	0.000	0.000	0.004	0.004	0.885
Colidextribacter	0.005	0.004	0.004	0.005	0.004	0.004	0.888
Family XIII UCG-001	0.004	0.004	0.004	0.003	0.002	0.004	0.891
Turicibacter	0.003	0.002	0.002	0.004	0.002	0.004	0.893
Mogibacterium	0.002	0.001	0.002	0.004	0.001	0.004	0.896
UBA1819	0.001	0.001	0.000	0.001	0.004	0.004	0.899
Lachnospiraceae FCS020 group	0.006	0.005	0.005	0.004	0.006	0.004	0.901
Bilophila	0.004	0.004	0.003	0.003	0.004	0.004	0.904
Phascolarctobacterium	0.004	0.003	0.004	0.003	0.005	0.003	0.906
Coprobacter	0.003	0.003	0.001	0.002	0.002	0.003	0.908
Candidatus Soleaferrea	0.001	0.000	0.001	0.003	0.001	0.003	0.911
Olsenella	0.001	0.001	0.001	0.003	0.001	0.003	0.913
Ligilactobacillus	0.002	0.001	0.003	0.004	0.002	0.003	0.915

Supplementary Table 2 **Genera frequencies in the enterotypes** (continued)

Taxa	Mean	m1	m2	m3	m4	diff	cdiff
Peptococcus	0.002	0.001	0.002	0.003	0.000	0.003	0.917
Lactococcus	0.004	0.003	0.002	0.003	0.004	0.003	0.920
[Clostridium] innocuum group	0.001	0.001	0.000	0.001	0.003	0.003	0.922
Lachnospiraceae UCG-001	0.003	0.003	0.003	0.002	0.002	0.003	0.924
Mitsuokella	0.001	0.000	0.001	0.002	0.000	0.003	0.926
Lachnospiraceae UCG-004	0.003	0.002	0.003	0.002	0.004	0.003	0.928
Succinivibrio	0.001	0.000	0.001	0.002	0.000	0.003	0.930
CAG-352	0.003	0.003	0.003	0.003	0.001	0.003	0.932
Oxalobacter	0.002	0.001	0.001	0.002	0.001	0.003	0.934
Megasphaera	0.002	0.001	0.002	0.003	0.002	0.003	0.936
Erysipelatoclostridium	0.001	0.001	0.000	0.000	0.002	0.003	0.938
Terrisporobacter	0.002	0.001	0.002	0.003	0.001	0.003	0.940
Eggerthella	0.001	0.000	0.000	0.000	0.002	0.003	0.942
Catenisphaera	0.001	0.000	0.001	0.002	0.000	0.003	0.944
Parasutterella	0.002	0.002	0.001	0.001	0.002	0.003	0.946
Adlercreutzia	0.001	0.002	0.000	0.000	0.002	0.003	0.947
UCG-009	0.002	0.001	0.001	0.002	0.001	0.003	0.949
Haemophilus	0.002	0.001	0.002	0.001	0.003	0.003	0.951
Fournierella	0.001	0.001	0.002	0.002	0.001	0.003	0.953
Methanosphaera	0.001	0.000	0.000	0.002	0.000	0.003	0.954
[Eubacterium] brachy group	0.001	0.001	0.000	0.000	0.002	0.002	0.956
Enterococcus	0.001	0.001	0.001	0.001	0.002	0.002	0.958
Allisonella	0.002	0.001	0.002	0.002	0.002	0.002	0.959
Actinomyces	0.001	0.000	0.001	0.001	0.002	0.002	0.961
Sellimonas	0.000	0.000	0.000	0.000	0.002	0.002	0.962
Coriobacteriaceae UCG-003	0.001	0.000	0.001	0.002	0.000	0.002	0.963
Holdemania	0.001	0.001	0.000	0.000	0.001	0.002	0.965
Libanicoccus	0.001	0.000	0.001	0.001	0.000	0.002	0.966
Veillonella	0.001	0.001	0.001	0.001	0.002	0.002	0.967
Defluviitaleaceae UCG-011	0.001	0.001	0.001	0.001	0.000	0.002	0.968
Anaerotruncus	0.001	0.001	0.000	0.000	0.002	0.002	0.970
FD2005	0.001	0.000	0.002	0.001	0.001	0.002	0.971
Granulicatella	0.000	0.000	0.000	0.000	0.001	0.002	0.972
Lachnospiraceae UCG-007	0.000	0.000	0.000	0.001	0.000	0.002	0.973
Limosilactobacillus	0.001	0.000	0.001	0.001	0.002	0.002	0.974
Negativibacillus	0.003	0.002	0.002	0.003	0.003	0.002	0.975
Lachnospiraceae NK3A20 group	0.000	0.000	0.001	0.001	0.000	0.001	0.976
Tyzzerella	0.000	0.000	0.000	0.000	0.001	0.001	0.977
Gemella	0.000	0.000	0.000	0.000	0.001	0.001	0.978
Sanguibacteroides	0.000	0.000	0.000	0.001	0.000	0.001	0.979
GCA-900 066 575	0.001	0.001	0.001	0.000	0.001	0.001	0.980

Supplementary Table 2 **Genera frequencies in the enterotypes** (continued)

Taxa	Mean	m1	m2	m3	m4	diff	cdiff
Cloacibacillus	0.000	0.000	0.000	0.001	0.000	0.001	0.980
Erysipelotrichaceae UCG-006	0.000	0.000	0.001	0.001	0.000	0.001	0.981
Eisenbergiella	0.000	0.000	0.000	0.000	0.001	0.001	0.982
Prevotella_7	0.001	0.000	0.001	0.001	0.001	0.001	0.983
[Eubacterium] fissicatena group	0.001	0.000	0.000	0.000	0.001	0.001	0.984
Oribacterium	0.000	0.000	0.000	0.001	0.000	0.001	0.984
Lachnospiraceae UCG-003	0.001	0.001	0.000	0.000	0.000	0.001	0.985
Gordonibacter	0.000	0.000	0.000	0.000	0.001	0.001	0.986
[Bacteroides] pectinophilus group	0.000	0.000	0.000	0.001	0.000	0.001	0.987
Shuttleworthia	0.000	0.000	0.000	0.000	0.001	0.001	0.987
Acidaminococcus	0.001	0.000	0.001	0.001	0.001	0.001	0.988
Rothia	0.001	0.000	0.001	0.000	0.001	0.001	0.989
Hungatella	0.000	0.000	0.000	0.000	0.001	0.001	0.989
Lachnospiraceae NK4B4 group	0.000	0.000	0.000	0.001	0.000	0.001	0.990
Leuconostoc	0.001	0.000	0.000	0.001	0.001	0.001	0.991
Phocea	0.000	0.000	0.000	0.000	0.001	0.001	0.991
Latilactobacillus	0.000	0.000	0.000	0.000	0.001	0.001	0.992
Lactobacillus	0.001	0.000	0.001	0.001	0.001	0.001	0.993
UCG-004	0.000	0.000	0.000	0.001	0.000	0.001	0.993
DTU089	0.000	0.000	0.000	0.000	0.001	0.001	0.994
Klebsiella	0.000	0.000	0.000	0.001	0.001	0.001	0.994
Coprobacillus	0.000	0.000	0.000	0.000	0.001	0.001	0.995
Merdibacter	0.000	0.000	0.000	0.000	0.001	0.001	0.995
Lacticaseibacillus	0.000	0.000	0.000	0.000	0.001	0.001	0.996
Ruminiclostridium	0.000	0.000	0.000	0.000	0.000	0.001	0.996
Weissella	0.001	0.000	0.000	0.001	0.001	0.001	0.997
Prevotellaceae NK3B31 group	0.000	0.000	0.000	0.001	0.000	0.001	0.997
Oscillospira	0.001	0.001	0.000	0.000	0.001	0.001	0.998
Christensenella	0.000	0.000	0.000	0.000	0.000	0.001	0.998
Victivallis	0.000	0.000	0.000	0.000	0.000	0.001	0.998
Paludicola	0.001	0.000	0.000	0.001	0.000	0.001	0.999
Asteroleplasma	0.000	0.000	0.000	0.000	0.000	0.001	0.999
Lactiplantibacillus	0.000	0.000	0.000	0.000	0.001	0.001	0.999
Megamonas	0.000	0.000	0.000	0.000	0.000	0.000	1.000
Enterobacter	0.000	0.000	0.000	0.000	0.000	0.000	1.000
